# Boosting cytosine base editing in potato through synergistic optimization

**DOI:** 10.1093/hr/uhag122

**Published:** 2026-04-07

**Authors:** Yan Zhang, Lumin Zhang, Jiuzhou Deng, Huiying Zhou, Enle Xiao, Guangtao Zhu, Chunzhi Zhang, Kaiyuan Chen

**Affiliations:** State Key Laboratory of Genome and Multi-omics Technologies, Shenzhen Branch, Guangdong Laboratory of Lingnan Modern Agriculture, Key Laboratory of Synthetic Biology, Ministry of Agriculture and Rural Affairs, Agricultural Genomics Institute at Shenzhen, Chinese Academy of Agricultural Sciences, Shenzhen 518120, China; School of Life Sciences, Henan University, Kaifeng 475004, China; Shenzhen Research Institute of Henan University, Shenzhen 518000, China; State Key Laboratory of Genome and Multi-omics Technologies, Shenzhen Branch, Guangdong Laboratory of Lingnan Modern Agriculture, Key Laboratory of Synthetic Biology, Ministry of Agriculture and Rural Affairs, Agricultural Genomics Institute at Shenzhen, Chinese Academy of Agricultural Sciences, Shenzhen 518120, China; Yunnan Key Laboratory of Potato Biology, School of Life Sciences, Yunnan Normal University, Kunming, Yunnan 650000, China; Yunnan Key Laboratory of Potato Biology, School of Life Sciences, Yunnan Normal University, Kunming, Yunnan 650000, China; Yunnan Key Laboratory of Potato Biology, School of Life Sciences, Yunnan Normal University, Kunming, Yunnan 650000, China; State Key Laboratory of Genome and Multi-omics Technologies, Shenzhen Branch, Guangdong Laboratory of Lingnan Modern Agriculture, Key Laboratory of Synthetic Biology, Ministry of Agriculture and Rural Affairs, Agricultural Genomics Institute at Shenzhen, Chinese Academy of Agricultural Sciences, Shenzhen 518120, China; State Key Laboratory of Genome and Multi-omics Technologies, Shenzhen Branch, Guangdong Laboratory of Lingnan Modern Agriculture, Key Laboratory of Synthetic Biology, Ministry of Agriculture and Rural Affairs, Agricultural Genomics Institute at Shenzhen, Chinese Academy of Agricultural Sciences, Shenzhen 518120, China

## Abstract

Base editing enables precise substitution of single nucleotides and is a promising technology for improvement of agronomic traits, including those of potato (*Solanum tuberosum*), the third most important food crop globally. Here, we developed efficient cytosine base editors (CBEs) for potato via multidimensional optimization. By evaluating several highly active cytidine deaminases, we identified two deaminases with high efficiency and distinct characteristics, which were used to construct CBEs for divergent editing applications. Furthermore, the editing efficiency of CBEs was significantly enhanced through several strategies: employing the *Arabidopsis thaliana RPS5A* promoter and the tobacco mosaic virus Ω enhancer to boost the expression of the editing reagents, fusing chromatin-modulating peptides, and co-expressing the human RNA m^6^A demethylase gene *hFTO* to enhance chromatin accessibility. In a hairy root assay, an efficient optimized base editor, RF-Sdd7-HNHN, elevated the average editing efficiency nearly 3-fold, from 26.8% (pre-optimization) to 76.7%. Using RF-Sdd7-HNHN, we achieved an 85.7% efficiency for simultaneous editing of target nucleotides in *ACETOLACTATE SYNTHASE 1* (*StALS1*) and *StALS2* and obtained homozygous mutant potato germplasm exhibiting herbicide resistance. In conclusion, this research established new and efficient CBEs for potato and offers insights and strategies for optimizing other CRISPR-based editing tools.

## Introduction

Potato (*Solanum tuberosum* L.), the most important noncereal crop worldwide, plays a significant role in ensuring global food security. Cultivated potato is an autotetraploid species (2*n* = 4*x* = 48) with a highly heterozygous genome, and this inherent genomic complexity has considerably impeded functional genomics research and genetic breeding progress. In recent years, with the proposition and implementation of diploid hybrid potato breeding, an increasing number of potato genomes have been sequenced and assembled, leading to a potato pangenome that, together with individual genomes, provides an important foundation for potato research and breeding [1–4]. To expedite the advancement of potato functional genomics, the development of efficient and functionally diverse genetic manipulation technologies for this species is essential.

As a mainly vegetatively propagated crop, potato has accumulated numerous deleterious mutations during its long-term domestication. Some of these deleterious mutations are, however, tightly linked with beneficial alleles, making them difficult to eliminate through traditional segregation via backcrossing [5, 6]. The advent of precision genome editing technologies, such as base editors (BEs), now makes it possible to rapidly repair such deleterious mutations. For example, base editing was used in tomato (*S. lycopersicum*) to directly correct a deleterious mutation in a floral regulator gene, yielding desirable crop traits [7]. Cytosine base editors (CBEs) and adenine base editors (ABEs) are the two most widely used base editing tools, typically comprising a fusion between a single-stranded DNA deaminase and a Cas nickase variant and catalyzing C-to-T and A-to-G base substitutions, respectively [8, 9]. Notably, only a few studies have focused on the application of CBEs and ABEs in potato, and the BE tools used in these studies lack in-depth optimization for this species, exhibit limited functional diversity, and require further improvement for efficiency [10–13].

In this study, we generated a series of CBE constructs tailored for potato and performed rounds of optimizations encompassing enhancements in the expression levels of CRISPR reagents and improvements in chromatin accessibility. Using these optimized CBE systems, we successfully generated herbicide-resistant potato germplasms. The optimizations significantly enhanced editing efficiency, providing robust tools for precise genome editing in potato and other species. These innovations are expected to accelerate research on plant gene function and crop breeding.

## Results

### Assessing the efficiency of multiple cytosine base editors in potato

**Figure 1:**
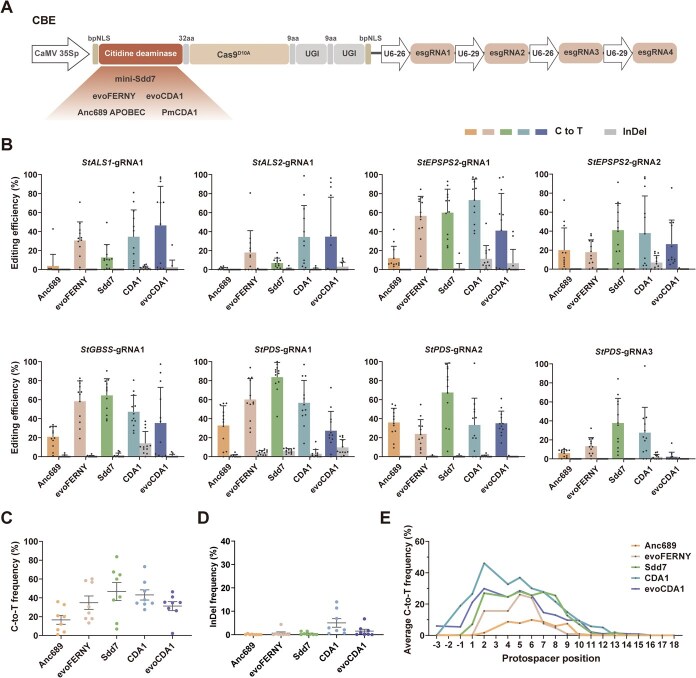
Assessment of multiple cytidine deaminases for the development of CBEs in potato. (A) Diagram of the CBE plasmids carrying one of multiple cytidine deaminase genes. (B) C-to-T editing efficiency and insertion/deletion (InDel) frequency obtained with each of five CBEs at eight endogenous target sites in the potato hairy root system. Values are means ± standard deviation (SD; *n* = 12 biological replicates). (C) Average C-to-T editing efficiency for each CBE across all eight target sites. Each circle represents the average editing efficiency of the indicated CBE at one target site. Values are means ± standard error of the mean (SEM; *n* = 8). (D) Average InDel frequency for each CBE across all eight target sites. Each circle represents the average InDel frequency of the indicated CBE at one target site. Values are means ± SEM (*n* = 8). (E) Position and mean C-to-T editing efficiency at all tested target sites relative to protospacer position for each of the five CBEs.

To develop robust CBEs for potato, we selected several cytidine deaminases reported to exhibit high efficiency in other species, including PmCDA1 [14], Anc689 APOBEC [15, 16], the two evolved cytidine deaminases evoCDA1 and evoFERNY [17, 18], and mini-Sdd7 [19]. Using the basic architecture of the BE4max base editor as a guide, we fused the sequence of each codon-optimized deaminase, uracil glycosylase inhibitor (UGI), and bipartite nuclear localization signal (bpNLS) in-frame with Cas9^D10A^ to generate five base editors, designated as CDA1, evoCDA1, evoFERNY, Anc689, and Sdd7 (Figs 1A and S1).

To rapidly test the efficiency of the five CBEs in potato as a proxy for their potential performance in stably transformed plants, we employed *Agrobacterium rhizogenes*-mediated stable genetic transformation of hairy roots to assess editing efficiency. We designed eight gRNAs targeting endogenous potato genes, with each employing an enhanced sgRNA scaffold (esgRNA) to enhance its expression [20] (Fig. 1B, Supplementary Tables S1 and S2). For each CBE plasmid, we obtained 12 independent transgenic hairy roots to evaluate editing performance at each test locus by high-throughput sequencing.

Analysis of the sequencing data revealed that these five CBEs could each generate C-to-T edits at all eight loci; notably, Sdd7 achieved the highest editing efficiency at five loci (37.7%–83.7%) (Fig. 1B, Supplementary Table S3). When examining editing efficiency across all targeted sites, Sdd7 also exhibited the highest editing efficiency of all five CBEs (46.7%), followed by CDA1 (43.1%), evoFERNY (34.8%), evoCDA1 (31.2%), and Anc689 (16.6%) (Fig. 1C). The InDel frequency induced by the use of Sdd7 (0.44%) was lower than that obtained with CDA1 (5.01%), evoCDA1 (1.51%), and evoFERNY (0.63%) (Fig. 1D and Supplementary Table S4). We also checked the editing windows of these CBEs. CDA1 showed high editing efficiency across a broad window (C_−1_–C_12_; counting the PAM as positions 21–23), while evoCDA1 also displayed a similarly wide editing window (C_−3_–C_10_). The editing window of Sdd7 was relatively narrower still (C_1_–C_10_), followed by those of evoFERNY and Anc689 (Figs 1E and S2).

Based on these results, we identified two efficient CBEs (Sdd7 and CDA1) with distinct characteristics. Sdd7 exhibits the highest editing efficiency, a low InDel frequency, and a relatively narrow editing window, making it suitable for precise base editing. In contrast, CDA1 maintains high efficiency across a broader editing window but has a higher InDel frequency, rendering it more appropriate for editing scenarios with low precision requirements and wide editing windows, such as gene saturation mutagenesis. To further enhance their editing efficiency, we first selected CDA1 as the test platform to evaluate the efficacy of subsequent optimization strategies.

### Using the *AtRPS5A* promoter, the TMV **Ω** enhancer, and RNA m^6^A demethylase hFTO synergistically improves editing efficiency

**Figure 2:**
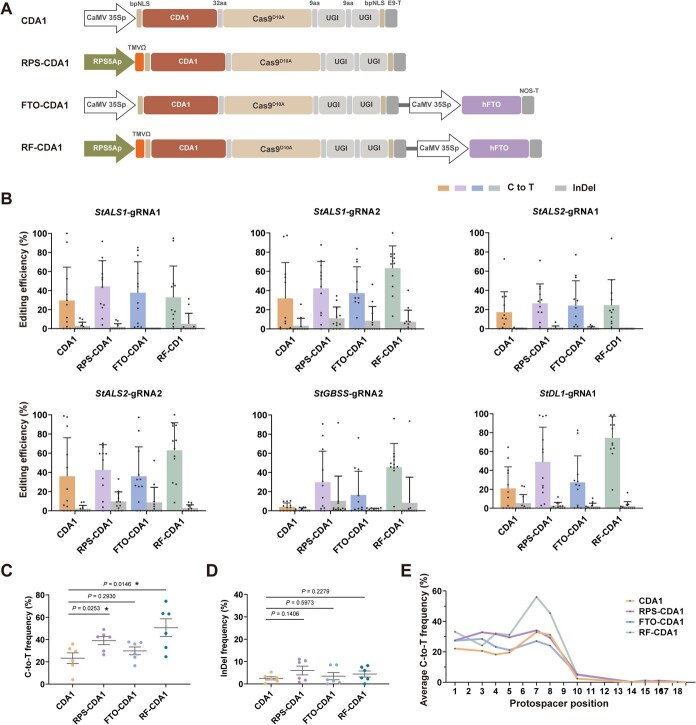
The use of the *AtRPS5A* promoter, the TMV Ω enhancer, and hFTO improves base editing efficiency. (A) Diagrams of the CBE plasmids with different promoters driving *CDA1-Cas9^D10A^* or co-expressing *hFTO*. *RPS5Ap*, *RPS5A* promoter from *A. thaliana*; TMV Ω, tobacco mosaic virus (TMV) Ω enhancer; hFTO, human RNA m^6^A demethylase FTO (Fat mass and obesity-associated protein). (B) C-to-T editing efficiency and InDel frequency of the four CBE plasmids at six endogenous sites in the potato hairy root system. Values are means ± SD (*n* = 12 biological replicates). (C) Average C-to-T editing efficiency for each CBE across all six target sites. Each circle represents the mean editing efficiency of the indicated CBE at one target site. Values are means ± SEM (*n* = 6). Statistical analysis was performed using Student’s *t*-test (^*^P < 0.05). (D) Average InDel frequency for each CBE across all six target sites. Each circle represents the mean InDel frequency for the indicated CBE at one target site. Values are means ± SEM (*n* = 6). Statistical analysis was performed using Student’s *t*-test. (E) Position and mean C-to-T editing efficiency at all tested target sites relative to protospacer position for each of the four CBEs.

Methods commonly used to improve the efficiency of the CRISPR system include improving the enzymatic activity of Cas, elevating the expression level of CRISPR reagents, and elevating chromatin accessibility, among others. According to reports, both the *Arabidopsis RPS5A* promoter and the Ω enhancer from tobacco mosaic virus (TMV) can enhance gene expression and improve the editing efficiency of CRISPR systems [21–24]. To enhance the expression of the cassette encoding the fusion between Cas9^D10A^ and the deaminase, we employed the *AtRPS5A* promoter and the TMV Ω enhancer in place of the cauliflower mosaic virus (CaMV) 35S promoter, yielding RPS-CDA1 (Fig. 2A). Additionally, a recent study showed that heterologous expression of *hFTO* in rice (*Oryza sativa*) and soybean (*Glycine max*) synergistically improved chromatin accessibility and upregulated *Cas* expression, leading to a higher efficiency for Cas9 and prime editing [25]. To test whether hFTO can enhance the efficiency of CBEs in potato, we modified the CDA1 plasmid by adding an expression cassette for *hFTO*, yielding the plasmid FTO-CDA1; in addition, we replaced the CaMV 35S promoter driving *CDA1-Cas9^D10A^* from FTO-CDA1 with the *Arabidopsis RPS5A* promoter, resulting in the RF-CDA1 plasmid (Fig. 2A).

We designed six gRNAs, each targeting a different locus in the potato genome for evaluation of each of these four CBE plasmids. Following the transformation of potato hairy roots and high-throughput sequencing, we detected enhanced editing efficiency at all sites when using RPS-CDA1 and FTO-CDA1 relative to CDA1, while RF-CDA1, integrating the two improvement strategies, further boosted the editing efficiency at all tested loci (Fig. 2B and Supplementary Table S5). When calculating the average editing efficiency of each CBE across all test loci, we observed the highest average editing efficiency (50.6%) from RF-CDA1, followed by RPS-CDA1 (39.0%) and FTO-CDA1 (29.8%), whereas CDA1 only achieved an editing efficiency of 23.2% (Fig. 2C). Notably, while they enhanced base editing efficiency, the three CBE systems incorporating either or both optimization strategies did not cause a significant rise in InDel frequency (Fig. 2D and Supplementary Table S6). Subsequently, we analyzed the editing windows of the four CBEs. The editing windows of the three optimized CBEs remained largely unchanged relative to that of CDA1, but the editing efficiency within these windows was higher (Figs 2E and S3). Taken together, these results demonstrate that both the *AtRPS5A* promoter and hFTO can enhance the editing efficiency of CDA1, and their combination exerts a synergistic effect that further boosts the efficiency of CDA1 without generating significantly more InDels.

### Chromatin-modulating peptides enhance the editing efficiency of CBEs and expand the editing window

**Figure 3:**
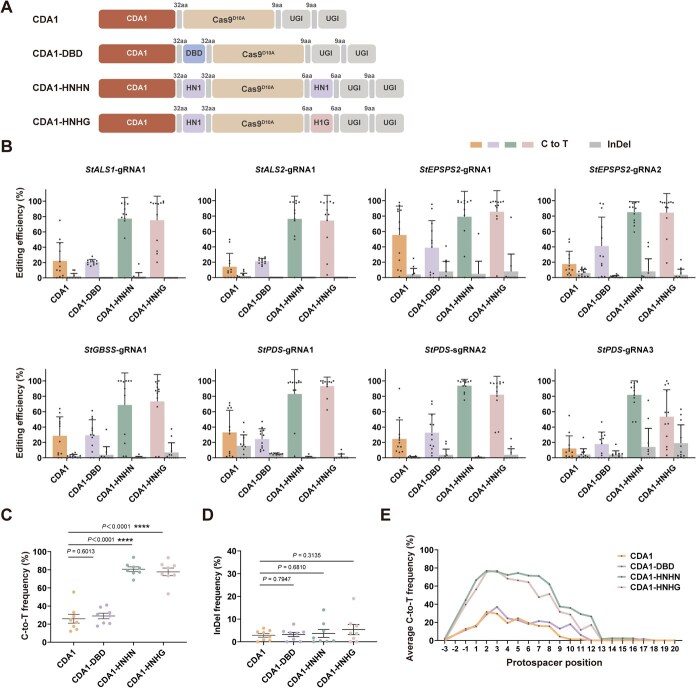
Adding a DBD and CMPs further enhances base editing efficiency. (A) Diagrams of the CBE plasmids carrying the single-stranded DBD of yeast Rad51 or CMPs. HN1, high mobility group protein HMGN1; H1G, human histone H1 central globular domain. (B) C-to-T editing efficiency and InDel frequency of four CBE plasmids at eight endogenous target sites in the potato hairy root system. Values are means ± SD (*n* = 12 biological replicates). (C) Average C-to-T editing efficiency of each CBE across all eight target sites. Each circle represents the mean editing efficiency of the indicated CBE at one target site. Values are means ± SEM (*n* = 8). Statistical analysis was performed using Student’s *t*-test (^*^P < 0.05, ^**^P < 0.01, ^***^P < 0.001, ^****^P < 0.0001). (D) Average InDel frequency of each CBE across all eight target sites. Each circle represents the mean InDel frequency of the indicated CBE at one target site. Values are means ± SEM (*n* = 8). Statistical analysis was performed using Student’s *t*-test. (E) Position and mean C-to-T editing efficiency at all tested target sites relative to protospacer position.

The cleavage activity of Cas nucleases is generally positively correlated with chromatin accessibility. Studies conducted in mammalian cells have demonstrated that the use of chromatin-modulating peptides (CMPs) can reshape chromatin states, thereby influencing CRISPR editing efficiency [26–28]. However, it remains unknown whether these CMPs can raise chromatin accessibility and improve base editing efficiency in plants. We, therefore, constructed the plasmids CDA1-HNHN and CDA1-HNHG by fusing CMPs from high mobility group protein HMGN1 (hereafter abbreviated as HN1) and the central globular domain of human histone H1 (hereafter abbreviated as H1G) in different combinations to the N terminus and C terminus of Cas9^D10A^ (Fig. 3A). Additionally, we fused the previously reported single-stranded DNA-binding domain (DBD) from yeast Rad51 to the CDA1-Cas9^D10A^ fusion protein, as this domain was shown to enhance editing efficiency and broaden the editing window [29, 30]; this resulted in plasmid CDA1-DBD (Fig. 3A). We transformed potato hairy roots with each plasmid and evaluated editing efficiency at eight target sites using multiplexed editing.

Analysis of high-throughput sequencing data revealed a modest rise in efficiency with CDA1-DBD (17.8%–41.0%) at the five sites tested, relative to that for CDA1 (12.1%–55.4%), while CDA1-HNHN (68.6%–93.8%) and CDA1-HNHG (53.4%–93.3%) produced significant gains in efficiency at all test loci (Fig. 3B and Supplementary Table S7). When looking at the average editing efficiency across the eight loci tested, CDA1-HNHN (80.6%) and CDA1-HNHG (77.7%) outperformed CDA1 (25.9%) by more than 3-fold, whereas the commonly used CDA1-DBD (29.0%) produced only a modest improvement relative to CDA1 (Fig. 3C). InDel frequency analysis at each site indicated that CDA1-HNHN and CDA1-HNHG enhance C-to-T editing efficiency without significantly raising InDel frequency (Fig. 3D and Supplementary Table S8). Moreover, the editing window of CDA1-DBD was expanded relative to that of CDA1 from C_−1_–C_9_ to C_−1_–C_11_, while the editing windows of CDA1-HNHN and CDA1-HNHG were extended to C_−1_–C_12_, along with significant concurrent improvements in efficiency within these windows (Figs 3E and S4). Collectively, these results demonstrate that modulating chromatin accessibility through CMP fusion can markedly improve both the editing efficiency and editing window of CBEs.

### Developing herbicide-resistant potato germplasms with optimized CBEs

**Figure 4:**
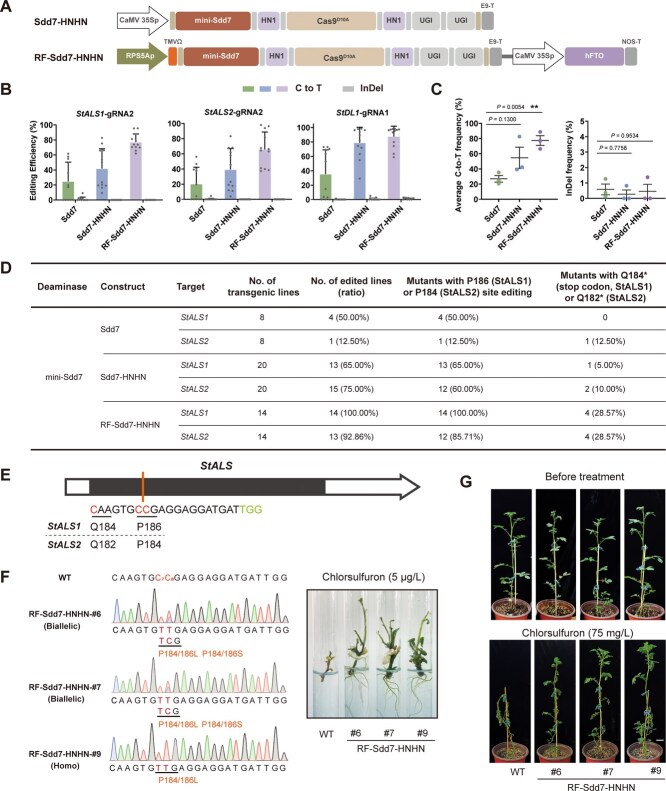
Optimized mini-Sdd7-based CBEs enable efficient C-to-T editing in both hairy roots and transgenic lines. (A) Diagrams of the optimized CBEs based on mini-Sdd7. (B) C-to-T editing efficiency and InDel frequency of three mini-Sdd7-based CBEs at three endogenous sites in the potato hairy root system. Values are means ± SD (*n* = 12 biological replicates). (C) Average C-to-T editing efficiency and InDel frequency of each CBE across all three target sites. Each circle represents the mean editing efficiency of the indicated CBE at one target site. Values are means ± SEM (*n* = 3). Statistical analysis was performed using Student’s *t*-test (^*^P < 0.05, ^**^P < 0.01). (D) Editing efficiency of CBEs based on the deaminase mini-Sdd7 in stably transformed transgenic potato lines. (E) Diagram of two ALS homologs (*StALS1* and *StALS2*) in potato and the cytosine bases targeted by the gRNA designed against *StALS1* and *StALS2*. (F) Sequencing chromatograms of three transgenic mutant lines (left) and growth status of these lines on MS medium containing 5 μg/l chlorsulfuron (right). The wild type (WT) was used as a control. (G) Representative photographs of transgenic lines and wild-type controls before (top) and after (bottom) spray treatment with 75 mg/l chlorsulfuron (at least three plants were treated per genotype). Scale bar, 5 cm.

Based on the above results of CDA1 optimization, we constructed the Sdd7-HNHN and RF-Sdd7-HNHN plasmids using another efficient deaminase, mini-Sdd7 (Fig. 4A). To compare the efficiency of the two optimized base editors with that of Sdd7, we targeted three endogenous loci in the potato genome via the hairy root transformation system. Sdd7 on its own showed low editing efficiency at the three loci (20.3%–35.2%), whereas the optimized Sdd7-HNHN and RF-Sdd7-HNHN systems were associated with greater efficiency at all loci tested (Fig. 4B and Supplementary Table S9). Specifically, RF-Sdd7-HNHN achieved the highest editing efficiency (65.6%–87.4%), raising editing efficiency by an average of 2.9-fold (76.7%) compared with that of Sdd7 (Figs 4C and S5). Notably, the InDel frequency remained at a low level, comparable with that of Sdd7 (Fig. 4C and Supplementary Table S10). These results further confirm that the optimization approaches used in this study confer excellent enhancing effects and have minimal effects on InDel frequency.

Creating herbicide-resistant germplasms has become an important direction in crop breeding. Acetolactate synthase (ALS) is a critical enzyme in branched-chain amino acid biosynthesis, and mutations at specific amino acid residues in this enzyme can confer herbicide resistance [31]. In potato, mutations introduced at the P186 site of StALS1 or the P184 site of StALS2 can confer resistance to sulfonylurea herbicides [12]. For targeted editing of these sites, we employed two base editors based on the deaminase PmCDA1 (CDA1, RF-CDA1) and three base editors based on mini-Sdd7 (Sdd7, Sdd7-HNHN, RF-Sdd7-HNHN), and designed a single gRNA that targets these sites in *StALS1* and *StALS2* simultaneously (Fig. 4D and E, Supplementary Tables S11 and S12). Through *A. tumefaciens*-mediated transformation with these plasmids, numerous stable transgenic lines were obtained.

High-throughput sequencing of the target sites determined that the base editors based on CDA1 and RF-CDA1 result in high editing efficiency (Supplementary Table S11). However, their wide editing window introduced unintended C-to-T substitutions at the codons encoding Q184 (StALS1) and Q182 (StALS2), in turn introducing premature stop codons. Moreover, the high InDel frequency exhibited by CDA1 and RF-CDA1 led to null alleles of *StALS1* and *StALS2*, with these two factors collectively causing abnormal growth in many transgenic plants (Supplementary Fig. S6 and Supplementary Table S11). By contrast, RF-Sdd7-HNHN exhibited high efficiency, low InDel frequency, and a narrower editing window, allowing precise editing of the codons encoding the target proline residues. In fact, 100% of the T_0_ lines harbored mutations at P186 (StALS1), and 85.7% of the lines had mutations at P184 (StALS2) (Fig. 4D and Supplementary Table S12). In addition, RF-Sdd7-HNHN showed high specificity with no off-target effects detected in the mutants (Supplementary Table S13).

We chose three homozygous or biallelic mutant lines for herbicide resistance assays. On culture medium containing 5 μg/l chlorsulfuron, apical buds excised from the three mutants developed roots and grew normally, while those from the wild-type controls were unable to form roots (Fig. 4F). When grown in a greenhouse, the mutants did not exhibit any clear phenotypic differences from the wild type. One week after being sprayed with chlorsulfuron, the mutants still grew normally, while the controls had wilted and died (Fig. 4G). In summary, our optimized RF-Sdd7-HNHN CBE shows high editing efficiency and a narrow editing window, and induces a low InDel frequency in both hairy roots and stable transgenic plants, enabling the efficient creation of herbicide-resistant potato germplasms through targeted editing of *StALS*.

## Discussion

As the world’s third most important food crop, the potato lags behind major staple crops like rice, wheat (*Triticum aestivum*), and maize (*Zea mays*) in both functional genomics and genetic manipulation technologies. In this study, we developed efficient CBEs by testing multiple high-performance cytosine deaminases, tailored to potato-specific characteristics. Furthermore, these CBEs were comprehensively optimized at multiple levels. Specifically, we incorporated the *Arabidopsis RPS5A* promoter and the TMV Ω enhancer to boost the expression of CRISPR reagents; to improve chromatin accessibility, we fused CMPs to the deaminase-Cas9^D10A^ fusion protein and co-expressed *hFTO*. Co-expression of *hFTO* further elevated the expression of deaminase-Cas9^D10A^ (Supplementary Fig. S7). These optimizations significantly enhanced editing efficiency and broadened the activity window.

Based on the properties of different deaminases, we developed specialized tools for potato. For instance, the deaminase mini-Sdd7 showed high efficiency, a narrow editing window, and a low InDel frequency, making it ideal for high-precision editing that demands exact editing products. In this study, mini-Sdd7-based CBEs are more suitable for the targeted editing of *StALS*. However, PmCDA1-based CBEs exhibit high efficiency across a broad editing window and a higher InDel frequency, making them suitable for applications such as saturation mutagenesis, for which a broader window is desired and precision is less critical.

We targeted the two *StALS* genes with the optimized base editor RF-Sdd7-HNHN and successfully developed herbicide-resistant potato germplasms, which could significantly reduce investment in weed control during potato production. Additionally, the combination of co-editing *ALS* and other genes of interest with herbicide screening has become an important approach for generating nontransgenic edited plants [32, 33], and our study provides an efficient tool for applying this method to more species.

While the development of CBEs and the associated optimizations presented in this study were primarily focused on potato, the various optimization strategies possess a certain degree of universality across species. Indeed, they are equally applicable for optimizing similar tools such as ABEs, prime editors (PEs), and other types of genome editing tools, and also serve as a reference for precise editing in species with relatively limited research progress. Potato genomics has advanced rapidly in recent years, as has research progress on diploid hybrid potato. The lag in functional genomics may be mitigated with increasingly efficient genetic manipulation tools.

## Methods

### Plant materials

The diploid potato accession ‘01–58’ was used in this study. 01–58 is a self-compatible potato clone with red skin and red flesh, derived from the cross between *S. tuberosum* group Stenotomum CIP700235 and E172 (carrying the S-locus inhibitor gene) [4, 34]. Potato plants were cultured on MS20 (Murashige and Skoog medium with 20 g/l sucrose) solid medium at 22°C under a 16-h light/8-h dark photoperiod. Three- to 4-week-old tissue-cultured plants were employed for *Agrobacterium*-mediated transformation. Tissue-cultured plants were transplanted to the greenhouse in soil and maintained under the same photoperiod and temperature conditions as those for tissue-cultured plants.

### Vector construction

To construct efficient CBE vectors for potato, the BE4max base editor was used as a guide for determining which elements to add to the CBE and in which order. The full-length coding sequences of *Anc689 APOBEC* and *evoFERNY* were obtained from previous studies [16, 18]. The coding sequences of the cytidine deaminase genes *mini-Sdd7*, *PmCDA1*, and *evoCDA1* were codon optimized for expression in potato and synthesized commercially (GenScript, Nanjing, China). These cytidine deaminase genes were individually inserted into the pENTR11-Cas9^D10A^ plasmid (digested with SacI) in-frame and upstream of the sequence encoding Cas9^D10A^ to produce entry plasmids for CBEs (pENTR11-Anc689, pENTR11-evoFERNY, pENTR11-Sdd7, pENTR11-CDA1, and pENTR11-evoCDA1). The five resulting entry plasmids were then individually recombined with the pCAMBIA2300-attRuv vector via a Gateway LR reaction to generate the CBE vectors Anc689, evoFERNY, Sdd7, CDA1, and evoCDA1, respectively.

To construct the vector harboring the *Arabidopsis RPS5A* promoter and *hFTO*, the *AtRPS5A* promoter was amplified by PCR from *Arabidopsis thaliana* (Col-0 ecotype) genomic DNA and the resulting amplicon was fused with the TMV Ω enhancer by overlap extension PCR. The CaMV 35S promoter in the CDA1 vector was replaced with the *AtRPS5Apro*-TMVΩ fragment, resulting in the RPS-CDA1 plasmid. The *hFTO* sequence codon optimized for potato was synthesized by GenScript (Nanjing, China). The full-length *hFTO* sequence was cloned between the CaMV 35S promoter and the *Nos* terminator, and the resulting fragment was inserted at the KpnI restriction site of the CDA1 and RPS-CDA1 plasmids to produce FTO-CDA1 and RF-CDA1, respectively.

To construct vectors carrying HN1 and H1G, the nucleotide sequences encoding HN1 and H1G codon optimized for potato were synthesized by GenScript. Subsequently, the HN1 sequence was cloned in-frame and upstream and downstream of the sequence encoding Cas9^D10A^ in pENTR11-CDA1, while the HN1 and H1G sequences were cloned in-frame and upstream (HN1) or downstream (H1G) of the *Cas9^D10A^* sequence, leading to the plasmids pENTR11-CDA1-HNHN and pENTR11-CDA1-HNHG, respectively. The sequence encoding the DBD of Rad51 was amplified from the vector pABE8e-D-SpRY and cloned into the pENTR11-Cas9^D10A^ plasmid to produce the entry vector pENTR11-Cas9^D10A^-DBD. Finally, the entry vectors were individually recombined with the pCAMBIA2300-attRuv vector via LR recombination to generate the final plasmids. The construction method for the vectors carrying *Sdd7* was similar to that for the *CDA1* series. Multiple gRNA expression cassettes were assembled by Golden Gate cloning as previously reported [35]. All primers used for vector construction are listed in Supplementary Table S14.

### *Agrobacterium*-mediated transformation

*Agrobacterium rhizogenes*-mediated hairy root transformation was performed as previously described [36]. *A. tumefaciens*-mediated stable transformation was performed with reference to the hairy root transformation protocol with the following modifications. Inoculated potato explants were placed on MS20 solid medium for 2 days in the dark at 22°C. After co-incubation of explants with *Agrobacterium* cultures, explants were transferred to MS20 medium containing 2 mg/l zeatin and 50 mg/l kanamycin sulfate. The medium was changed every 2 weeks, and the explants were maintained under the same growth conditions as those for tissue-cultured plants.

### Mutation detection and off-target detection

Genomic DNA was isolated from hairy roots or leaves using the cetyltrimethylammonium bromide method. The target gene fragments were amplified via PCR using Phanta Max Super-Fidelity DNA Polymerase (Vazyme, #P505). The PCR products were analyzed by Sanger sequencing or high-throughput sequencing on the Hi-TOM platform for mutation detection [37]. To detect off-target effects in mutant lines, we used Cas-OFFinder (http://www.rgenome.net/cas-offinder/) to predict potential off-target sites, and then employed Hi-TOM high-throughput sequencing to examine the off-target editing of these sites.

### Herbicide resistance test for *Stals* mutants

To test the herbicide resistance of *StALS*-edited potato plants under *in vitro* conditions, the apical buds of wild-type controls and mutant plants were transferred to MS20 medium containing 5 μg/l chlorsulfuron (Sigma-Aldrich). The explants were maintained at 22°C under a 16-h light/8-h dark photoperiod for about 1 month, during which rooting and growth of the explants were monitored. For plants grown in the greenhouse, 7- to 8-week-old plants (post-transplantation into the greenhouse) were sprayed with a solution containing 75 mg/l chlorsulfuron and 0.1% (v/v) Tween 20. Each treatment comprised at least three biological replicates, and plant phenotypes were evaluated about 1 week after treatment.

### Statistical analysis

Statistical significance was analyzed with GraphPad Prism 9.5 software using the two-tailed unpaired Student’s *t*-test. *P* < 0.05 was considered statistically significant.

## Supplementary Material

Web_Material_uhag122

## Data Availability

All data supporting the conclusions of this study are available in the article and supplementary information. The plasmids encoding the optimized CBEs are available to the scientific community upon request to the corresponding author.

## References

[1] Cheng L, Wang N, Bao Z. et al. Leveraging a phased pangenome for haplotype design of hybrid potato. Nature. 2025;640:408–17 39843749 10.1038/s41586-024-08476-9 PMC11981936

[2] Sun H, Tusso S, Dent CI. et al. The phased pan-genome of tetraploid European potato. Nature. 2025;642:389–97 40240601 10.1038/s41586-025-08843-0 PMC12158759

[3] Tang D, Jia Y, Zhang J. et al. Genome evolution and diversity of wild and cultivated potatoes. Nature. 2022;606:535–41 35676481 10.1038/s41586-022-04822-x PMC9200641

[4] Zhang C, Yang Z, Tang D. et al. Genome design of hybrid potato. Cell. 2021;184:3873–3883.e12 34171306 10.1016/j.cell.2021.06.006

[5] Wu Y, Li D, Hu Y. et al. Phylogenomic discovery of deleterious mutations facilitates hybrid potato breeding. Cell. 2023;186:2313–2328.e15 37146612 10.1016/j.cell.2023.04.008

[6] Zhang C, Wang P, Tang D. et al. The genetic basis of inbreeding depression in potato. Nat Genet. 2019;51:374–8 30643248 10.1038/s41588-018-0319-1

[7] Glaus AN, Brechet M, Swinnen G. et al. Repairing a deleterious domestication variant in a floral regulator gene of tomato by base editing. Nat Genet. 2025;57:231–41 39747596 10.1038/s41588-024-02026-9

[8] Pacesa M, Pelea O, Jinek M. Past, present, and future of CRISPR genome editing technologies. Cell. 2024;187:1076–100 38428389 10.1016/j.cell.2024.01.042

[9] Wang JY, Doudna JA. CRISPR technology: a decade of genome editing is only the beginning. Science. 2023;379:eadd8643 36656942 10.1126/science.add8643

[10] Chincinska IA, Miklaszewska M, Sołtys-Kalina D. Recent advances and challenges in potato improvement using CRISPR/Cas genome editing. Planta. 2022;257:25 36562862 10.1007/s00425-022-04054-3 PMC9789015

[11] Veillet F, Kermarrec MP, Chauvin L. et al. CRISPR-induced indels and base editing using the *Staphylococcus aureus* Cas9 in potato. PLoS One. 2020;15:e0235942 32804931 10.1371/journal.pone.0235942 PMC7430721

[12] Veillet F, Perrot L, Chauvin L. et al. Transgene-free genome editing in tomato and potato plants using *Agrobacterium*-mediated delivery of a CRISPR/Cas9 Cytidine Base editor. Int J Mol Sci. 2019;20:402 30669298 10.3390/ijms20020402 PMC6358797

[13] Zong Y, Wang Y, Li C. et al. Precise base editing in rice, wheat and maize with a Cas9-cytidine deaminase fusion. Nat Biotechnol. 2017;35:438–40 28244994 10.1038/nbt.3811

[14] Nishida K, Arazoe T, Yachie N. et al. Targeted nucleotide editing using hybrid prokaryotic and vertebrate adaptive immune systems. Science. 2016;353:aaf8729 27492474 10.1126/science.aaf8729

[15] Koblan LW, Doman JL, Wilson C. et al. Improving cytidine and adenine base editors by expression optimization and ancestral reconstruction. Nat Biotechnol. 2018;36:843–6 29813047 10.1038/nbt.4172 PMC6126947

[16] Wang M, Wang Z, Mao Y. et al. Optimizing base editors for improved efficiency and expanded editing scope in rice. Plant Biotechnol J. 2019;17:1697–9 30963683 10.1111/pbi.13124 PMC6686124

[17] Thuronyi BW, Koblan LW, Levy JM. et al. Continuous evolution of base editors with expanded target compatibility and improved activity. Nat Biotechnol. 2019;37:1070–9 31332326 10.1038/s41587-019-0193-0 PMC6728210

[18] Zeng D, Liu T, Tan J. et al. PhieCBEs: plant high-efficiency cytidine base editors with expanded target range. Mol Plant. 2020;13:1666–9 33152517 10.1016/j.molp.2020.11.001

[19] Huang J, Lin Q, Fei H. et al. Discovery of deaminase functions by structure-based protein clustering. Cell. 2023a;186:3182–3195.e14 37379837 10.1016/j.cell.2023.05.041

[20] Chen B, Gilbert LA, Cimini BA. et al. Dynamic imaging of genomic loci in living human cells by an optimized CRISPR/Cas system. Cell. 2013;155:1479–91 24360272 10.1016/j.cell.2013.12.001 PMC3918502

[21] Gallie DR, Kado CI. A translational enhancer derived from tobacco mosaic virus is functionally equivalent to a Shine-Dalgarno sequence. Proc Natl Acad Sci U S A. 1989;86:129–32 2643095 10.1073/pnas.86.1.129 PMC286417

[22] Kang BC, Yun JY, Kim ST. et al. Precision genome engineering through adenine base editing in plants. Nat Plants. 2018;4:427–31 29867128 10.1038/s41477-018-0178-x

[23] Peng F, Zhang W, Zeng W. et al. Gene targeting in *Arabidopsis* via an all-in-one strategy that uses a translational enhancer to aid Cas9 expression. Plant Biotechnol J. 2020;18:892–4 31553828 10.1111/pbi.13265 PMC7061861

[24] Tsutsui H, Higashiyama T. pKAMA-ITACHI vectors for highly efficient CRISPR/Cas9-mediated gene knockout in *Arabidopsis thaliana*. Plant Cell Physiol. 2017;58:46–56 27856772 10.1093/pcp/pcw191 PMC5444565

[25] Bai M, Lin W, Peng C. et al. Expressing a human RNA demethylase as an assister improves gene-editing efficiency in plants. Mol Plant. 2024;17:363–6 38368507 10.1016/j.molp.2024.02.010

[26] Ding X, Seebeck T, Feng Y. et al. Improving CRISPR-Cas9 genome editing efficiency by fusion with chromatin-modulating peptides. CRISPR J. 2019;2:51–63 31021236 10.1089/crispr.2018.0036

[27] Park SJ, Jeong TY, Shin SK. et al. Targeted mutagenesis in mouse cells and embryos using an enhanced prime editor. Genome Biol. 2021;22:170 34082781 10.1186/s13059-021-02389-w PMC8173820

[28] Yang C, Ma Z, Wang K. et al. HMGN1 enhances CRISPR-directed dual-function A-to-G and C-to-G base editing. Nat Commun. 2023;14:2430 37105976 10.1038/s41467-023-38193-2 PMC10140177

[29] Xue N, Liu X, Zhang D. et al. Improving adenine and dual base editors through introduction of TadA-8e and Rad51DBD. Nat Commun. 2023;14:1224 36869044 10.1038/s41467-023-36887-1 PMC9984408

[30] Zhang X, Chen L, Zhu B. et al. Increasing the efficiency and targeting range of cytidine base editors through fusion of a single-stranded DNA-binding protein domain. Nat Cell Biol. 2020;22:740–50 32393889 10.1038/s41556-020-0518-8

[31] Luo Q, Liu YG. Breeding herbicide-resistant rice using CRISPR-Cas gene editing and other technologies. Plant Commun. 2025;6:101172 39397365 10.1016/j.xplc.2024.101172 PMC11897542

[32] Huang X, Jia H, Xu J. et al. Transgene-free genome editing of vegetatively propagated and perennial plant species in the T0 generation via a co-editing strategy. Nat Plants. 2023b;9:1591–7 37723203 10.1038/s41477-023-01520-y

[33] Lu Y, Naren T, Qiao D. et al. One-step generation of prime-edited transgene-free rice. Plant Commun. 2025;6:101227 39709522 10.1016/j.xplc.2024.101227 PMC12010367

[34] Du H, Zhai Z, Pu J. et al. Two tandem R2R3 MYB transcription factor genes cooperatively regulate anthocyanin accumulation in potato tuber flesh. Plant Biotechnol J. 2025;23:1521–34 39887502 10.1111/pbi.14602 PMC12018810

[35] Ma X, Zhang Q, Zhu Q. et al. A robust CRISPR/Cas9 system for convenient, high-efficiency multiplex genome editing in monocot and dicot plants. Mol Plant. 2015;8:1274–84 25917172 10.1016/j.molp.2015.04.007

[36] Butler NM, Jansky SH, Jiang J. First-generation genome editing in potato using hairy root transformation. Plant Biotechnol J. 2020;18:2201–9 32170801 10.1111/pbi.13376 PMC7589382

[37] Liu Q, Wang C, Jiao X. et al. Hi-TOM: a platform for high-throughput tracking of mutations induced by CRISPR/Cas systems. Sci China Life Sci. 2019;62:1–7 30446870 10.1007/s11427-018-9402-9

