# Supplementary material for: Boosting cytosine base editing in potato through synergistic optimization
Source: Hortic Res. 2026 Apr 7;13(8):uhag122. doi: 10.1093/hr/uhag122 (PMC13395515; doi:10.1093/hr/uhag122)
Supplement: Web_Material_uhag122 [file web_material_uhag122.zip › Revised Supplemental Figure 1-7 and Table 1-2 0302 .pdf]

Supplemental Information

Boosting Cytosine Base Editing in Potato through Synergistic Optimization

Yan Zhang[1,5], Lumin Zhang[2,3,5], Jiuzhou Deng[1,5], Huiying Zhou[4], Enle Xiao[4], Guangtao Zhu[4], Chunzhi Zhang[1,*], Kaiyuan Chen[1,*]

[1]State Key Laboratory of Genome and Multi-omics Technologies, Shenzhen Branch, Guangdong Laboratory of Lingnan Modern Agriculture, Key Laboratory of Synthetic Biology, Ministry of Agriculture and Rural Affairs, Agricultural Genomics Institute at Shenzhen, Chinese Academy of Agricultural Sciences, Shenzhen 518120, China

[2]School of Life Sciences, Henan University, Kaifeng 475004, China.

[3]Shenzhen Research Institute of Henan University, Shenzhen 518000, China

[4]Yunnan Key Laboratory of Potato Biology, School of Life Sciences, Yunnan Normal University, Kunming, Yunnan 650000, China

[5]These authors contributed equally to this article

*Correspondence: Kaiyuan Chen (chenkaiyuan@caas.cn), Chunzhi Zhang (zhangchunzhi01@caas.cn); Tel: +860755-23250159; Fax: +860755-23251430

>mini-Sdd7

GAAGGTGGAGGACCAGGTGCTGTTCCTGAGGGTGGAGATGGACCTCCAGCTGTTCCAGCTGAAGAGGTTGAAAGACTTAGGGGTGAGTTGCCTCCACCTGTTGTTCCAGGTACTGGACAAAAGACACATGGAAGGTGGATTGGTCCTGATGGAAGAGTTAGGGCTATTGTTTCTGGAAGAGATGAAGATGCTGCTCTTGTTCATGCTCAATTGGCTGCTAAGGGAATTCCAGATGAACCTACTAGGAATTCAGATGTTGAGCAAAAACTTGCTGCTCATATGGTTGCTAATGGTATTAGACATGTTACTTTGGTTATTAACCATAGGCCATGTAGAGGATTTGATGATTCTTGTGATACACTTGTTCCAATTATTTTGCCTGAGGGTTGTACTCTTACAGTTCATGGTCAAACTGATAAAGGAATGAGAGTTAGGGTTAGATATACAGGTGGAGCTAGGCCTTGGTGGTCA

>PmCDA1

ACAGACGCTGAATATGTTAGAATCCATGAAAAACTGGATATCTATACATTTAAGAAGCAGTTCTTCAATAACAAAAAGTCAGTATCTCACAGATGCTATGTCCTGTTCGAACTCAAGAGAAGAGGAGAAAGGCGGGCCTGTTTCTGGGGGTACGCGGTTAATAAACCCCAGTCCGGGACCGAGAGGGGGATTCACGCCGAGATCTTTTCAATTAGGAAGGTTGAAGAGTATCTTCGCGACAATCCCGGTCAGTTCACAATTAACTGGTACAGCTCCTGGAGCCCTTGCGCTGATTGCGCCGAGAAAATACTCGAATGGTACAACCAGGAGTTGAGAGGCAATGGCCACACTCTCAAGATTTGGGCTTGCAAACTTTACTACGAGAAGAACGCGAGAAATCAGATTGGCTTGTGGAACCTCAGGGACAACGGGGTCGGGTTGAATGTTATGGTGTCCGAACATTACCAGTGCTGTAGAAAGATCTTCATTCAGTCCAGTCACAATCAGCTGAACGAGAACAGATGGCTGGAGAAAACACTGAAACGGGCAGAGAAAAGGCGCTCAGAGCTGAGTATCATGATCCAGGTCAAAATCCTGCATACAACCAAAAGCCCGGCTGTA

>evoCDA1

ACAGACGCTGAATATGTTAGAATCCATGAAAAACTGGATATCTATACATTTAAGAAGCAGTTCTCTAATAACAAAAAGTCAGTATCTCACAGATGCTATGTCCTGTTCGAACTCAAGAGAAGAGGAGAAAGGCGGGCCTGTTTCTGGGGGTACGCGGTTAATAAACCCCAGTCCGGGACCGAGAGGGGGATTCACGCCGAGATCTTTTCAATTAGGAAGGTTGAAGAGTATCTTCGCGACAATCCCGGTCAGTTCACAATTAACTGGTACAGCTCCTGGAGCCCTTGCGCTGATTGCGCCGAGAAAATACTCGAATGGTACAACCAGGAGTTGAGAGGCAATGGCCACACTCTCAAGATTTGGGTTTGCAAACTTTACTACGAGAAGAACGCGAGAAATCAGATTGGCTTGTGGAACCTCAGGGACAACGGGGTCGGGTTGAATGTTATGGTGTCCGAACATTACCAGTGCTGTAGAAAGATCTTCATTCAGTCCAGTCACAATCAGCTGAACGAGAACAGATGGCTGGAGAAAACACTGAAACGGGCAGAGAAAAGGCGCTCAGAGCTGAGTATCATGTTCCAGGTCAAAATCCTGCATACAACCAAAAGCCCGGCTGTA

>HN1

ATGCCTAAGAGAAAAGTTTCTTCAGCTGAAGGAGCTGCTAAGGAAGAACCAAAAAGAAGGTCTGCTAGGCTTTCAGCTAAGCCTCCAGCTAAAGTTGAGGCTAAGCCTAAGAAAGCTGCTGCTAAGGATAAATCTTCAGATAAGAAGGTTCAAACTAAGGGAAAAAGAGGTGCTAAGGGAAAACAAGCTGAAGTTGCTAATCAAGAAACAAAAGAGGATTTGCCTGCTGAAAATGGAGAGACTAAGACAGAGGAATCTCCAGCTTCAGATGAAGCTGGAGAAAAGGAAGCTAAATCTGAT

>H1G

TCAACTGATCATCCAAAGTACTCTGATATGATTGTTGCTGCTATTCAAGCTGAAAAGAATAGAGCTGGTTCTTCAAGGCAATCTATTCAAAAGTACATTAAGTCACATTACAAAGTTGGAGAGAATGCTGATTCTCAAATTAAGCTTTCAATTAAGAGATTGGTTACTACAGGTGTTCTTAAGCA

AACAAAAGGAGTTGGTGCTTCTGGATCATTTAGGTTGGCTAAGTCAGATGAACCA

>hFTO

ATGAAAAGAACTCCTACAGCTGAAGAGAGAGAGAGGGAAGCTAAGAAACTTAGGCTTTTG
GAAGAGTTGGAAGATACTTGGCTTCCTTATTTGACACCAAAGGATGATGAGTTTTATCAACA
ATGGCAACTTAAGTACCCAAAGCTTATTTTGAGAGAAGCTTCTTCAGTTTCTGAAGAGTTGC
ATAAAGAAGTTCAAGAGGCTTTTCTTACTTTGCATAAGCATGGTTGTCTTTTTAGAGATTTG
GTTAGGATTCAAGGAAAAGATCTTTTGACTCCTGTTTCAAGAATTCTTATTGGAAACCCAGG
TTGTACATACAAGTACCTTAACACTAGGTTGTTTACAGTTCCTTGGCCAGTTAAGGGTTCAA
ACATTAAGCATACTGAGGCTGAAATTGCTGCTGCTTGTGAAACATTTCTTAAGTTGAACGAT
TACCTTCAAATTGAGACTATTCAAGCTCTTGAAGAGTTGGCTGCTAAGGAAAAAGCTAATG
AGGATGCTGTTCCTTTGTGTATGTCTGCTGATTTTCCAAGAGTTGGAATGGGTTCTTCATACA
ACGGTCAAGATGAAGTTGATATTAAGTCAAGGGCTGCTTACAACGTTACACTTTTGAACTTC
ATGGATCCTCAAAAGATGCCATACCTTAAGGAAGAGCCTTATTTTGGAATGGGAAAGATGG
CTGTTTCTTGGCATCATGATGAAAATTTGGTTGATAGATCAGCTGTTGCTGTTTATTCTTATTC
ATGTGAGGGACCTGAAGAGGAATCTGAAGATGATTCACATCTTGAGGGTAGGGATCCAGAT
ATTTGGCATGTTGGATTCAAGATTTCATGGGATATTGAAACTCCTGGTCTTGCTATTCCATTG
CATCAAGGAGATTGTTACTTCATGCTTGATGATTTGAATGCTACTCATCAACATTGTGTTTTG
GCTGGTTCTCAACCTAGATTTTCTTCAACACATAGGGTTGCTGAATGTTCAACTGGAACACT
TGATTACATTTTGCAAAGATGTCAACTTGCTTTGCAAAACGTTTGTGATGATGTTGATAACG
ATGATGTTTCTCTTAAGTCATTTGAACCAGCTGTTTTGAAGCAAGGAGAGGAAATTCATAAT
GAGGTTGAATTTGAGTGGTTGAGACAATTCTGGTTCCAAGGAAACAGATATAGGAAGTGTA
CTGATTGGTGGTGTCAACCTATGGCTCAACTTGAAGCTTTGTGGAAGAAAATGGAGGGTGT
TACAAATGCTGTTCTTCATGAAGTTAAGAGAGAGGGATTGCCAGTTGAACAAAGGAATGAG
ATTCTTACTGCTATTCTTGCTTCTTTGACAGCTAGACAAAATTTGAGAAGGGAATGGCATGC
TAGGTGTCAATCAAGAATTGCTAGGACTCTTCCTGCTGATCAAAAACCTGAATGTAGACCAT
ATTGGGAGAAGGATGATGCTTCTATGCCTCTTCCATTCGATTTGACTGATATTGTTTCAGAAT
TGAGGGGTCAACTTTTGGAGGCTAAACCA

Supplemental Figure 1. The potato codon-optimized nucleotide sequences that encode mini-Sdd7, PmCDA1, evoCDA1, HN1, H1G and hFTO.

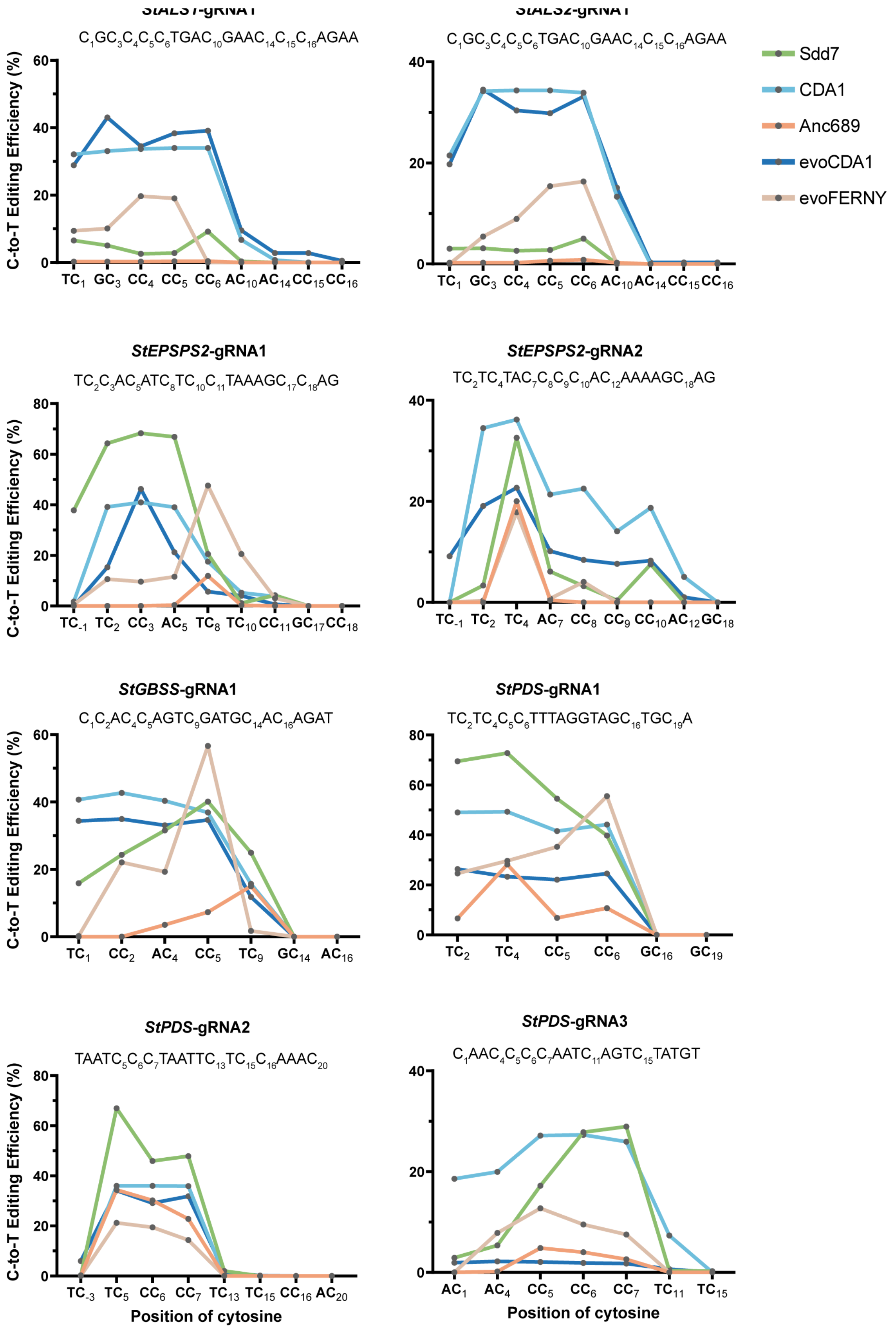

Supplemental Figure 2. The C-to-T editing activity windows of five cytosine base editors at each target site.

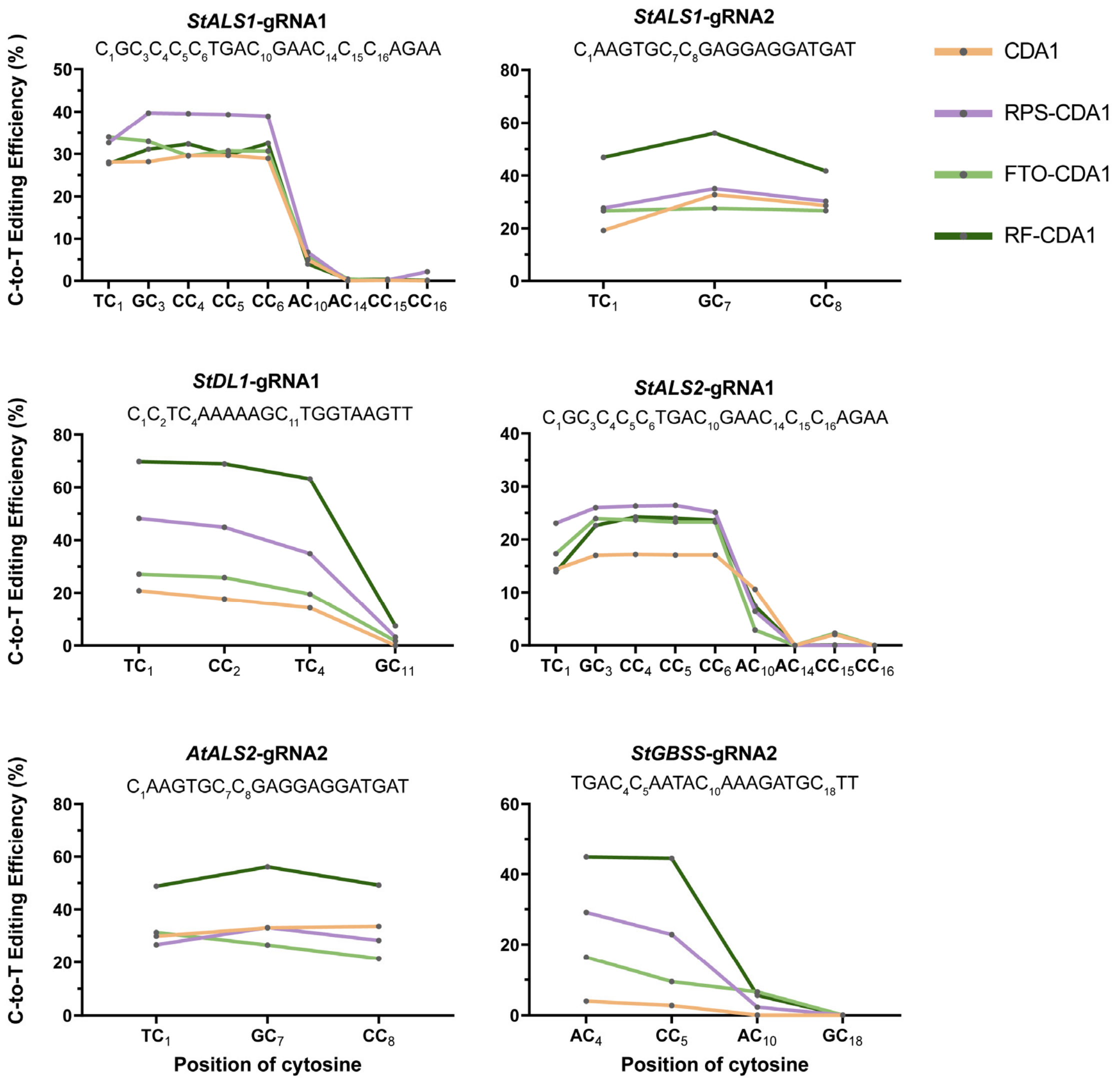

Supplemental Figure 3. The C-to-T editing activity windows of the four cytosine base editors CDA1, RPS-CDA1, FTO-CDA1 and RF-CDA1 at each target site.

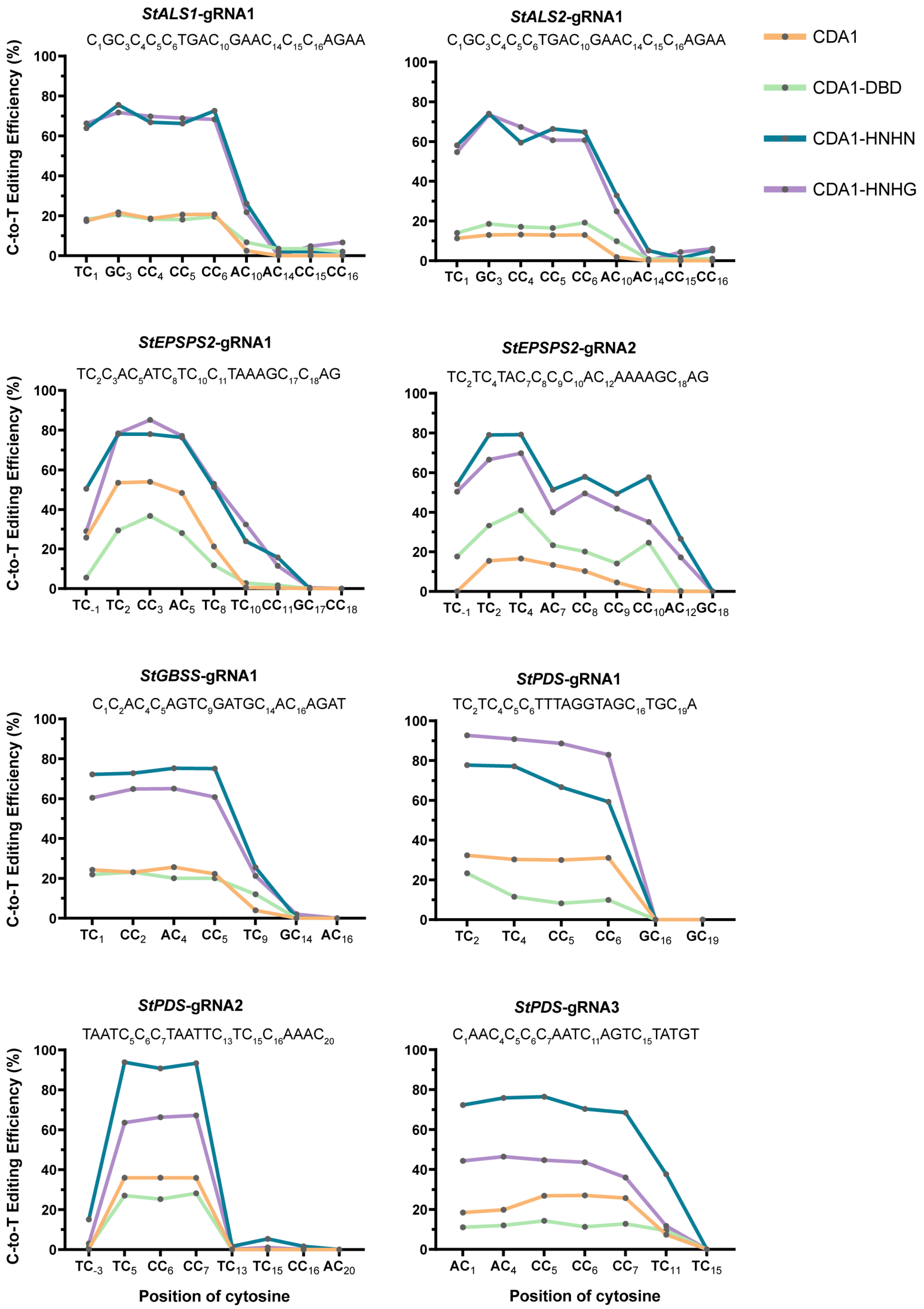

Supplemental Figure 4. The C-to-T editing activity windows of the four cytosine base editors CDA1, CDA1-DBD, CDA1-HNHN and CDA1-HNHG at each target site.

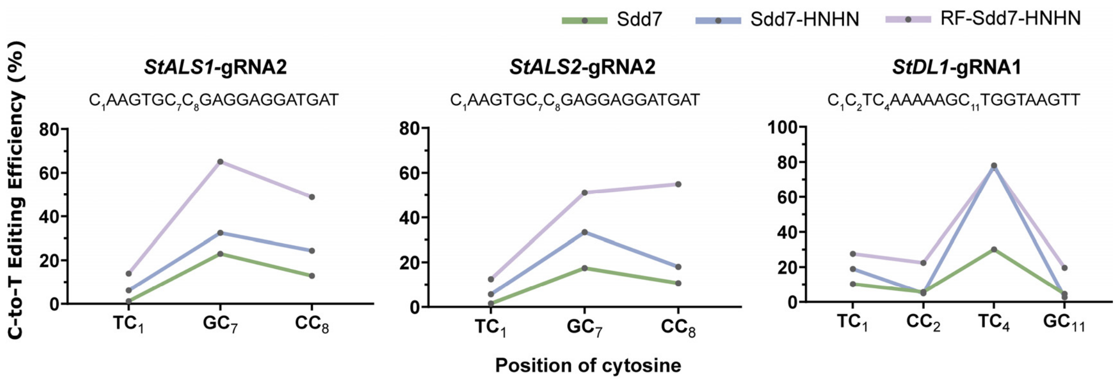

Supplemental Figure 5. The C-to-T editing activity windows of the three mini-Sdd7-based cytosine base editors Sdd7, Sdd7-HNHN and RF-Sdd7-HNHN at each target site.

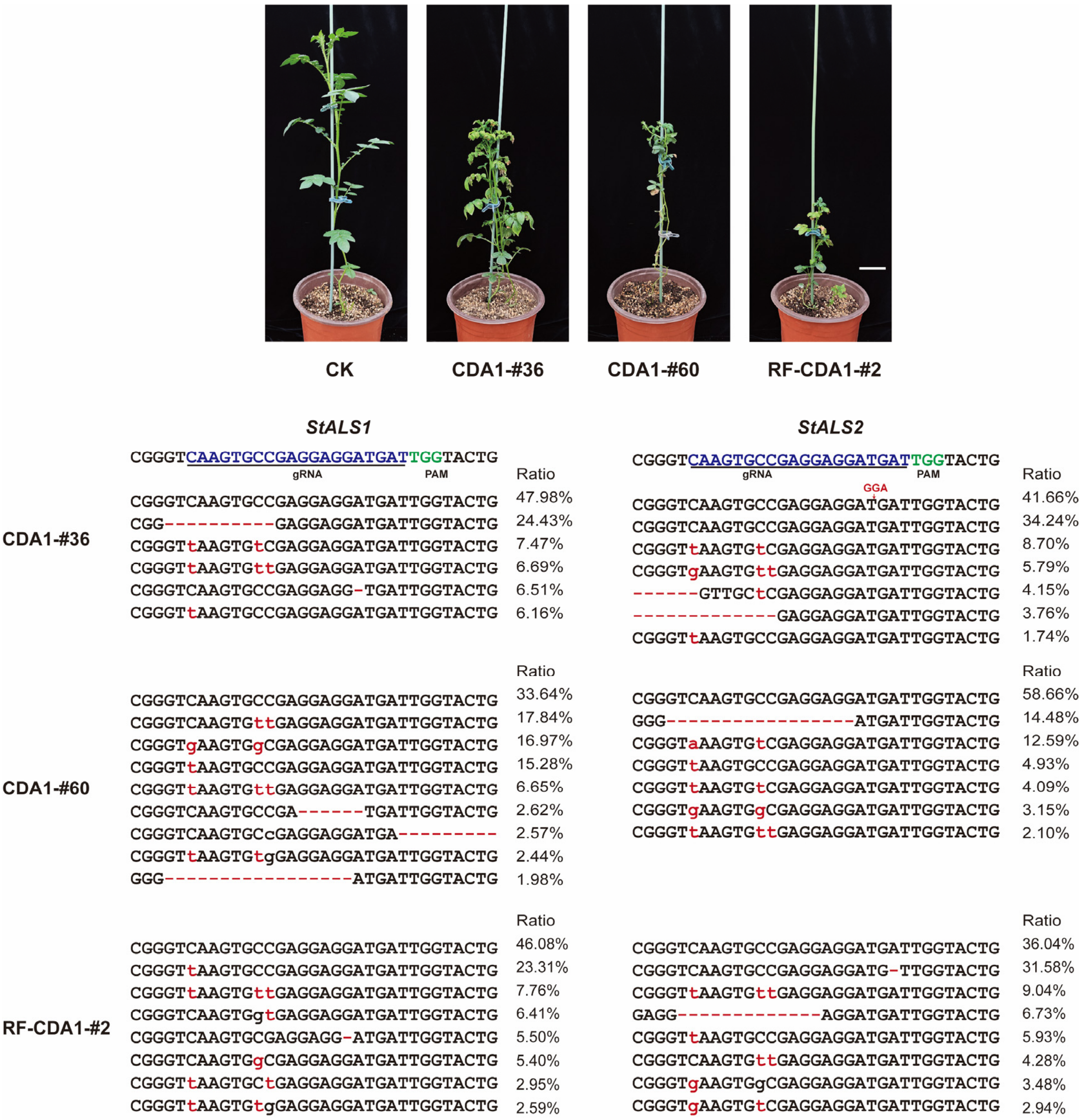

Supplemental Figure 6. Phenotypic images and genotyping of plants showing developmental defects in *Stals* mutant lines generated by CDA1 and RF-CDA1. CDA1 transgenic line with unmutated target genes served as control (CK). Bar = 5 cm

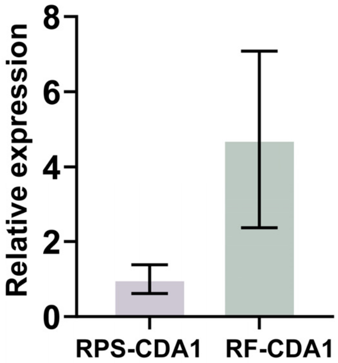

Supplemental Figure 7. Expression of *CDA1* in RPS-CDA1 and RF-CDA1 transgenic lines. Three biological replicates were performed, and the error bars represent the standard errors of three biological replicates.

Supplemental Table 1. List of target sequences used in this study.

Target	Gene ID	gRNA	Sequence (5'-3')	PAM
StALS1	Soltu.DM.03G005810	gRNA1	CGCCCCTGACGAACCCAGAA	AGG
		gRNA2	CAAGTGCCGAGGAGGATGAT	TGG
StALS2	Soltu.DM.07G023080	gRNA1	CGCCCCTGACGAACCCAGAA	AGG
		gRNA2	CAAGTGCCGAGGAGGATGAT	TGG
StGBSS	Soltu.DM.08G030230	gRNA1	CCACCAGTCGATGCACAGAT	TGG
		gRNA2	TGACCAATACAAAGATGCTT	GGG
StEPSPS2	Soltu.DM.05G020760	gRNA1	TCCACATCTCCTAAAGCCAG	TGG
		gRNA2	TCTCTACCCCACAAAAGCAG	GGG
StPDS	Soltu.DM.03G037550	gRNA1	TCTCCCTTTAGGTAGCTGCA	TGG
		gRNA2	TAATCCCTAATTCTCCAAAC	AGG
		gRNA3	CAACCCCAATCAGTCTATGT	TGG
StDL1	Soltu.DM.08G030290	gRNA1	CCTCAAAAAGCTGGTAAGTT	CGG

Supplemental Table 2. The gRNA expression cassettes for multiplex base editing in this study.

Construct ID	gRNA expression cassettes
T1	U626p::*StALS*-esgRNA1-U629p::*StEPSPS2*-esgRNA1-U626p::*StPDS*-esgRNA1
T2	U626p::*StEPSPS2*-esgRNA2-U629p::*StGBSS*-esgRNA1-U626p::*StPDS*-esgRNA2-U629p::*StPDS*-esgRNA3
T3	U626p::*StALS*-esgRNA1-U629p::*StALS*-esgRNA2-U626p::*StGBSS*-esgRNA2-U629p::*StDL1*-esgRNA1
