# Supplementary material for: Boosting cytosine base editing in potato through synergistic optimization
Source: Hortic Res. 2026 Apr 7;13(8):uhag122. doi: 10.1093/hr/uhag122 (PMC13395515; doi:10.1093/hr/uhag122)
Supplement: Web_Material_uhag122 [file web_material_uhag122.zip › Revised Supplemental Table 3-14 .pdf]

Supplemental Table 3. The C-to-T editing efficiency of Anc689, evoFERNY, Sdd7, CDA1 and evoCDA1 in potato hairy roots

Target sites	CBE constructs	C-to-T editing efficiency in independent transgenic hairy roots											
		1	2	3	4	5	6	7	8	9	10	11	12
StALS1-gRNA1	Anc689	0.00%	0.00%	0.00%	0.00%	0.00%	0.00%	0.00%	0.00%	0.00%	42.75%	1.01%	1.04%
	evoFERNY	38.18%	29.65%	31.01%	32.83%	70.90%	14.01%	33.46%	41.71%	48.65%	2.31%	0.00%	24.09%
	Sdd7	5.04%	1.25%	11.39%	25.99%	49.47%	1.19%	9.76%	0.00%	11.26%	10.75%	15.89%	10.29%
	CDA1	40.82%	61.55%	0.00%	22.80%	3.90%	16.62%	45.26%	6.72%	9.06%	71.50%	54.01%	80.95%
	evoCDA1	2.33%	47.91%	92.77%	95.49%	0.00%	1.12%	6.66%	70.86%	70.74%	97.32%	1.37%	70.41%
StALS2-gRNA1	Anc689	1.24%	0.00%	2.07%	0.00%	0.00%	0.00%	0.00%	0.00%	1.28%	2.99%	0.00%	3.70%
	evoFERNY	3.45%	22.58%	28.10%	19.92%	80.67%	0.00%	15.17%	13.35%	0.00%	0.00%	33.02%	0.00%
	Sdd7	6.12%	1.41%	6.11%	3.66%	1.92%	9.08%	4.19%	0.00%	9.87%	17.82%	12.76%	9.57%
	CDA1	53.84%	25.20%	0.00%	9.80%	2.12%	29.91%	78.71%	6.76%	3.46%	63.96%	98.70%	39.84%
	evoCDA1	0.00%	0.00%	78.24%	91.87%	0.00%	0.00%	0.00%	25.85%	96.34%	87.00%	1.15%	35.86%
StEPSPS2-gRNA1	Anc689	12.04%	0.00%	42.26%	29.46%	5.99%	6.77%	5.90%	4.85%	5.55%	6.95%	3.45%	20.96%
	evoFERNY	43.28%	32.13%	71.89%	60.25%	84.51%	14.10%	75.77%	70.62%	44.14%	74.26%	55.58%	52.79%
	Sdd7	37.00%	72.63%	32.00%	25.05%	88.68%	92.91%	59.30%	23.25%	60.71%	76.32%	79.02%	72.47%
	CDA1	85.46%	47.43%	95.13%	84.70%	66.71%	38.04%	92.40%	40.94%	73.14%	96.27%	60.27%	97.34%
	evoCDA1	97.42%	58.59%	0.00%	1.15%	1.93%	41.47%	0.00%	0.00%	52.19%	84.13%	57.87%	96.64%
StEPSPS2-gRNA2	Anc689	2.85%	9.53%	12.01%	50.29%	45.82%	7.40%	0.00%	72.58%	17.42%	17.20%	5.29%	0.00%
	evoFERNY	1.42%	37.05%	11.00%	14.12%	7.05%	29.84%	29.47%	23.79%	35.34%	0.00%	6.38%	18.65%
	Sdd7	18.62%	70.31%	43.58%	59.87%	39.79%	50.36%	91.39%	25.76%	0.00%	5.77%	67.28%	19.01%
	CDA1	87.21%	0.00%	58.49%	11.46%	95.34%	6.09%	96.94%	0.00%	54.52%	37.24%	3.96%	3.82%
	evoCDA1	10.13%	8.27%	48.90%	46.04%	6.47%	85.51%	49.09%	4.92%	23.91%	9.63%	12.42%	10.36%
StGBSS-gRNA1	Anc689	31.78%	1.14%	33.56%	31.09%	18.94%	19.61%	31.35%	25.05%	11.25%	17.09%	3.98%	26.95%
	evoFERNY	58.07%	29.11%	65.70%	67.89%	79.82%	19.07%	73.61%	77.34%	36.19%	82.54%	68.08%	42.66%
	Sdd7	37.90%	73.07%	61.16%	43.46%	50.93%	71.19%	79.43%	39.54%	90.37%	76.49%	80.75%	69.37%
	CDA1	52.63%	37.63%	38.01%	80.50%	45.19%	64.97%	69.02%	24.82%	53.32%	40.21%	27.34%	32.88%
	evoCDA1	97.34%	60.60%	0.00%	1.19%	2.84%	36.99%	1.28%	0.00%	15.21%	79.53%	40.86%	88.41%
StPDS-gRNA1	Anc689	54.64%	56.03%	56.19%	0.00%	5.25%	42.99%	39.28%	46.77%	31.96%	4.44%	19.09%	36.46%
	evoFERNY	55.07%	82.70%	57.35%	80.41%	93.74%	30.01%	37.55%	83.40%	55.61%	64.39%	25.72%	56.37%
	Sdd7	77.16%	91.36%	97.15%	89.16%	78.61%	86.06%	42.22%	100.00%	87.62%	90.25%	93.50%	70.93%
	CDA1	43.99%	61.01%	54.74%	48.70%	13.89%	32.22%	85.00%	64.93%	43.21%	85.01%	96.95%	49.32%
	evoCDA1	36.47%	15.01%	22.67%	39.36%	25.21%	72.61%	2.41%	18.50%	22.76%	52.19%	0.00%	19.72%
StPDS-gRNA2	Anc689	13.18%	26.85%	55.31%	46.22%	53.99%	39.50%	9.30%	50.09%	42.41%	26.36%	34.86%	32.76%
	evoFERNY	4.01%	32.34%	19.73%	28.56%	18.37%	9.19%	54.85%	23.39%	7.47%	14.00%	32.06%	42.94%
	Sdd7	28.97%	67.13%	83.98%	80.87%	94.48%	97.32%	92.65%	29.66%	5.58%	56.76%	98.64%	73.80%
	CDA1	40.48%	0.00%	47.29%	19.69%	98.36%	5.91%	49.88%	0.00%	41.00%	55.46%	22.16%	19.51%
	evoCDA1	32.04%	44.70%	41.06%	61.15%	28.75%	51.57%	34.82%	14.36%	34.65%	29.74%	22.23%	25.41%
StPDS-gRNA3	Anc689	3.14%	5.97%	2.44%	10.79%	7.80%	8.44%	0.00%	6.85%	8.65%	9.04%	9.10%	1.12%
	evoFERNY	3.08%	10.25%	12.74%	12.74%	5.65%	16.35%	32.18%	20.80%	5.66%	0.00%	18.56%	21.19%
	Sdd7	18.00%	37.82%	46.43%	21.31%	57.62%	67.67%	84.24%	7.75%	3.91%	11.52%	60.53%	35.42%
	CDA1	43.64%	0.00%	29.70%	19.07%	97.77%	8.58%	7.44%	0.00%	42.60%	36.68%	23.02%	21.77%
	evoCDA1	1.84%	0.00%	0.00%	5.31%	0.00%	16.56%	0.00%	0.00%	2.44%	0.00%	0.00%	0.00%

Supplemental Table 4. The InDel frequency of Anc689, evoFERNY, Sdd7, CDA1 and evoCDA1 in potato hairy roots

Target sites	CBE constructs	The InDel frequency of CBEs in independent transgenic hairy roots											
		1	2	3	4	5	6	7	8	9	10	11	12
StALS1-gRNA1	Anc689	0.00%	0.00%	0.00%	0.00%	0.00%	0.00%	0.00%	0.00%	0.00%	0.00%	0.00%	0.00%
	evoFERNY	0.00%	0.00%	0.00%	0.00%	0.00%	0.00%	0.00%	0.00%	0.00%	0.00%	0.00%	0.00%
	Sdd7	0.00%	0.00%	0.00%	0.00%	0.00%	0.00%	0.00%	0.00%	0.00%	0.00%	0.00%	0.00%
	CDA1	1.59%	0.00%	0.00%	1.46%	1.95%	0.00%	4.83%	5.49%	2.01%	1.15%	0.00%	2.41%
	evoCDA1	0.00%	0.00%	0.00%	0.00%	0.00%	0.00%	0.00%	4.07%	25.93%	0.00%	0.00%	0.00%
StALS2-gRNA1	Anc689	0.00%	0.00%	0.00%	0.00%	0.00%	0.00%	0.00%	0.00%	0.00%	0.00%	0.00%	0.00%
	evoFERNY	0.00%	0.00%	0.00%	0.00%	0.00%	0.00%	1.14%	0.00%	0.00%	0.00%	0.00%	0.00%
	Sdd7	0.00%	0.00%	0.00%	0.00%	0.00%	0.00%	0.00%	0.00%	4.20%	0.00%	0.00%	0.00%
	CDA1	0.00%	0.00%	0.00%	0.00%	0.00%	0.00%	2.00%	3.97%	0.00%	0.00%	0.00%	0.00%
	evoCDA1	0.00%	0.00%	9.97%	0.00%	0.00%	0.00%	0.00%	11.91%	0.00%	7.22%	0.00%	8.36%
StEPSPS2-gRNA1	Anc689	0.00%	0.00%	0.00%	0.00%	0.00%	0.00%	0.00%	0.00%	0.00%	0.00%	0.00%	0.00%
	evoFERNY	0.00%	0.00%	0.00%	0.00%	0.00%	0.00%	0.00%	0.00%	1.25%	0.00%	0.00%	0.00%
	Sdd7	0.00%	0.00%	0.00%	0.00%	0.00%	0.00%	17.30%	0.00%	0.00%	0.00%	0.00%	0.00%
	CDA1	4.75%	20.90%	0.00%	4.36%	17.42%	19.85%	4.06%	48.64%	7.92%	1.64%	7.28%	0.00%
	evoCDA1	0.00%	35.39%	0.00%	0.00%	0.00%	0.00%	0.00%	0.00%	40.10%	0.00%	4.12%	1.02%
StEPSPS2-gRNA2	Anc689	0.00%	0.00%	0.00%	0.00%	0.00%	0.00%	0.00%	0.00%	0.00%	0.00%	0.00%	0.00%
	evoFERNY	0.00%	0.00%	0.00%	0.00%	0.00%	0.00%	0.00%	0.00%	0.00%	0.00%	0.00%	0.00%
	Sdd7	0.00%	0.00%	0.00%	0.00%	0.00%	0.00%	0.00%	0.00%	0.00%	0.00%	0.00%	0.00%
	CDA1	3.16%	0.00%	11.77%	8.18%	0.00%	1.93%	0.00%	0.00%	16.70%	5.73%	18.45%	16.98%
	evoCDA1	0.00%	0.00%	0.00%	0.00%	0.00%	0.00%	0.00%	0.00%	0.00%	0.00%	0.00%	1.21%
StGBSS-gRNA1	Anc689	0.00%	0.00%	0.00%	0.00%	0.00%	3.91%	1.82%	0.00%	0.00%	0.00%	0.00%	0.00%
	evoFERNY	0.00%	0.00%	0.00%	0.00%	0.00%	0.00%	0.00%	0.00%	0.00%	1.10%	0.00%	2.91%
	Sdd7	3.80%	6.21%	0.00%	0.00%	0.00%	0.00%	0.00%	1.01%	1.03%	0.00%	0.00%	5.33%
	CDA1	10.14%	8.07%	10.43%	3.84%	8.07%	11.62%	0.00%	33.75%	11.00%	3.14%	37.20%	30.46%
	evoCDA1	0.00%	0.00%	0.00%	0.00%	0.00%	0.00%	0.00%	0.00%	5.09%	0.00%	0.00%	3.77%
StPDS-gRNA1	Anc689	0.00%	1.52%	0.00%	0.00%	0.00%	0.00%	0.00%	0.00%	0.00%	1.04%	0.00%	0.00%
	evoFERNY	5.38%	4.08%	5.05%	2.14%	5.53%	6.42%	1.38%	4.11%	1.61%	1.59%	8.00%	6.94%
	Sdd7	8.56%	2.73%	6.07%	2.59%	8.63%	9.13%	4.02%	4.28%	7.85%	6.57%	7.76%	4.95%
	CDA1	1.08%	0.00%	0.00%	1.73%	3.72%	0.00%	7.77%	0.00%	3.14%	15.82%	0.00%	1.27%
	evoCDA1	22.12%	5.03%	4.78%	21.51%	4.61%	17.04%	0.00%	5.56%	10.48%	20.52%	1.27%	4.48%
StPDS-gRNA2	Anc689	0.00%	0.00%	0.00%	0.00%	0.00%	0.00%	0.00%	0.00%	0.00%	0.00%	0.00%	0.00%
	evoFERNY	0.00%	0.00%	0.00%	1.81%	0.00%	0.00%	0.00%	0.00%	0.00%	0.00%	0.00%	0.00%
	Sdd7	0.00%	0.00%	0.00%	0.00%	0.00%	0.00%	0.00%	0.00%	0.00%	0.00%	0.00%	2.64%
	CDA1	3.87%	0.00%	1.11%	0.00%	0.00%	0.00%	0.00%	0.00%	0.00%	0.00%	1.28%	1.23%
	evoCDA1	0.00%	0.00%	0.00%	0.00%	0.00%	0.00%	0.00%	0.00%	0.00%	0.00%	1.19%	0.00%
StPDS-gRNA3	Anc689	0.00%	0.00%	0.00%	0.00%	0.00%	0.00%	0.00%	0.00%	0.00%	0.00%	0.00%	0.00%
	evoFERNY	0.00%	0.00%	0.00%	0.00%	0.00%	0.00%	0.00%	0.00%	0.00%	0.00%	0.00%	0.00%
	Sdd7	0.00%	0.00%	0.00%	0.00%	0.00%	0.00%	0.00%	0.00%	0.00%	0.00%	0.00%	0.00%
	CDA1	1.25%	0.00%	7.12%	3.76%	0.00%	3.90%	2.13%	0.00%	0.00%	6.63%	0.00%	0.00%
	evoCDA1	0.00%	0.00%	0.00%	0.00%	0.00%	1.18%	0.00%	0.00%	0.00%	0.00%	0.00%	0.00%

Supplemental Table 5. The C-to-T editing efficiency of CDA1, RPS-CDA1, FTO-CDA1 and RF-CDA1 in potato hairy roots

Target sites	CBE constructs	C-to-T editing efficiency in independent transgenic hairy roots											
		1	2	3	4	5	6	7	8	9	10	11	12
StALS1-gRNA1	CDA1	35.88%	0.00%	7.09%	11.56%	0.00%	100.00%	2.14%	90.79%	25.11%	52.35%	29.82%	0.00%
	RPS-CDA1	26.06%	43.56%	10.41%	24.85%	70.87%	57.32%	53.49%	91.68%	74.58%	51.40%	3.91%	22.02%
	FTO-CDA1	5.61%	1.51%	26.89%	58.00%	43.99%	21.26%	76.97%	47.11%	2.17%	85.16%	1.62%	82.37%
	RF-CDA1	19.65%	94.94%	92.32%	2.03%	47.25%	0.00%	44.05%	15.02%	45.17%	18.02%	10.15%	5.15%
StALS2-gRNA1	CDA1	24.51%	10.93%	0.00%	1.32%	14.25%	72.72%	0.00%	33.66%	10.32%	33.30%	4.95%	0.00%
	RPS-CDA1	17.26%	22.94%	0.00%	43.84%	17.10%	35.90%	30.14%	41.64%	71.05%	28.54%	1.88%	6.48%
	FTO-CDA1	2.14%	0.00%	4.30%	30.77%	21.78%	29.24%	55.45%	12.93%	2.33%	53.56%	0.00%	77.02%
	RF-CDA1	18.90%	50.60%	94.10%	0.00%	7.49%	30.25%	16.99%	19.96%	37.26%	12.14%	1.72%	5.42%
StALS1-gRNA2	CDA1	96.28%	97.45%	0.00%	5.73%	7.87%	0.00%	1.30%	57.07%	1.76%	48.50%	53.70%	11.66%
	RPS-CDA1	66.06%	44.05%	70.89%	14.19%	4.31%	87.52%	16.73%	51.94%	59.03%	55.38%	0.00%	37.10%
	FTO-CDA1	27.38%	32.19%	0.00%	32.30%	83.48%	0.00%	72.45%	28.36%	40.84%	11.64%	70.07%	49.19%
	RF-CDA1	13.29%	78.03%	44.22%	55.43%	73.82%	67.79%	34.13%	70.91%	78.09%	75.51%	100.00%	68.31%
StALS2-gRNA2	CDA1	98.75%	88.27%	0.00%	5.81%	4.42%	0.00%	0.00%	58.54%	22.63%	45.14%	97.64%	10.51%
	RPS-CDA1	52.91%	67.95%	66.14%	9.32%	0.00%	59.35%	26.67%	59.64%	70.24%	33.70%	3.61%	59.56%
	FTO-CDA1	32.69%	32.48%	0.00%	25.40%	97.31%	0.00%	25.17%	26.21%	62.13%	9.06%	37.45%	83.36%
	RF-CDA1	8.50%	100.00%	56.87%	54.23%	64.89%	89.05%	27.72%	29.11%	73.39%	90.68%	88.37%	72.77%
StDL1-gRNA1	CDA1	17.12%	37.09%	0.00%	0.00%	53.21%	0.00%	0.00%	40.07%	2.68%	64.56%	24.74%	11.29%
	RPS-CDA1	72.32%	98.35%	97.04%	23.74%	4.32%	98.12%	31.96%	62.32%	16.61%	21.62%	2.80%	59.24%
	FTO-CDA1	1.09%	2.93%	24.89%	19.95%	82.36%	0.00%	24.09%	0.00%	35.73%	28.33%	29.20%	79.48%
	RF-CDA1	19.35%	98.69%	57.77%	63.40%	68.84%	88.18%	96.05%	67.01%	62.77%	84.07%	98.56%	88.20%
StGBSS-gRNA2	CDA1	3.55%	9.91%	0.00%	0.00%	5.05%	0.00%	0.00%	9.80%	0.00%	3.38%	5.61%	10.36%
	RPS-CDA1	0.00%	90.67%	64.28%	42.72%	2.13%	78.50%	13.64%	30.60%	5.11%	2.86%	0.00%	26.28%
	FTO-CDA1	0.00%	2.26%	3.53%	1.51%	9.40%	0.00%	43.24%	0.00%	15.81%	1.48%	44.61%	76.15%
	RF-CDA1	4.33%	45.83%	46.08%	41.96%	48.32%	49.57%	44.80%	59.56%	56.55%	52.87%	96.24%	0.00%

Supplemental Table 6. The InDel frequency of CDA1, RPS-CDA1, FTO-CDA1 and RF-CDA1 in potato hairy roots

Target sites	CBE constructs	The InDel frequency of CBEs in independent transgenic hairy roots											
		1	2	3	4	5	6	7	8	9	10	11	12
StALS1-gRNA1	CDA1	6.56%	0.00%	9.73%	2.17%	11.06%	0.00%	0.00%	0.00%	0.00%	3.20%	0.00%	0.00%
	RPS-CDA1	0.00%	9.14%	0.00%	0.00%	8.01%	0.00%	0.00%	0.00%	0.00%	3.44%	0.00%	0.00%
	FTO-CDA1	0.00%	0.00%	0.00%	0.00%	0.00%	0.00%	0.00%	0.00%	0.00%	0.00%	0.00%	0.00%
	RF-CDA1	0.00%	0.00%	0.00%	25.45%	1.67%	0.00%	1.99%	0.00%	0.00%	0.00%	0.00%	31.31%
StALS2-gRNA1	CDA1	0.00%	0.00%	0.00%	1.40%	0.00%	0.00%	0.00%	0.00%	0.00%	0.00%	0.00%	0.00%
	RPS-CDA1	0.00%	6.81%	0.00%	0.00%	0.00%	0.00%	0.00%	0.00%	0.00%	2.66%	0.00%	0.00%
	FTO-CDA1	0.00%	0.00%	0.00%	0.00%	0.00%	0.00%	0.00%	2.75%	0.00%	0.00%	0.00%	4.16%
	RF-CDA1	0.00%	0.00%	0.00%	0.00%	0.00%	0.00%	0.00%	0.00%	0.00%	0.00%	0.00%	0.00%
StALS1-gRNA2	CDA1	0.00%	0.00%	0.00%	0.00%	0.00%	0.00%	0.00%	11.68%	0.00%	25.67%	2.59%	0.00%
	RPS-CDA1	3.77%	34.37%	13.93%	21.42%	0.00%	12.49%	5.38%	6.35%	0.00%	0.00%	4.92%	29.97%
	FTO-CDA1	0.00%	28.55%	0.00%	0.00%	0.00%	0.00%	4.34%	0.00%	16.30%	46.44%	0.00%	6.65%
	RF-CDA1	0.00%	21.97%	0.00%	7.55%	5.08%	7.77%	40.26%	5.73%	0.00%	0.00%	0.00%	1.72%
StALS2-gRNA2	CDA1	0.00%	3.85%	0.00%	2.11%	0.00%	0.00%	0.00%	8.85%	0.00%	2.20%	0.00%	9.05%
	RPS-CDA1	2.70%	17.61%	13.10%	20.29%	0.00%	33.16%	0.00%	9.19%	5.41%	0.00%	5.09%	9.21%
	FTO-CDA1	0.00%	27.40%	0.00%	0.00%	0.00%	0.00%	9.64%	0.00%	6.81%	52.30%	1.92%	4.50%
	RF-CDA1	0.00%	0.00%	2.34%	6.20%	8.29%	0.00%	0.00%	8.90%	0.00%	0.00%	1.87%	5.12%
StDL1-gRNA1	CDA1	12.19%	23.54%	0.00%	0.00%	0.00%	0.00%	0.00%	23.07%	2.98%	0.00%	0.00%	2.08%
	RPS-CDA1	2.22%	0.00%	0.00%	5.61%	0.00%	0.00%	5.83%	2.19%	1.33%	11.91%	0.00%	1.63%
	FTO-CDA1	0.00%	0.00%	2.01%	0.00%	0.00%	0.00%	2.59%	0.00%	6.66%	1.11%	10.56%	0.00%
	RF-CDA1	0.00%	0.00%	0.00%	0.00%	3.96%	3.62%	0.00%	1.95%	16.40%	0.00%	0.00%	1.23%
StGBSS-gRNA2	CDA1	0.00%	0.00%	0.00%	1.72%	1.09%	0.00%	0.00%	2.78%	0.00%	3.56%	3.39%	3.80%
	RPS-CDA1	91.91%	2.73%	2.51%	1.41%	9.81%	0.00%	2.83%	1.08%	7.94%	1.05%	1.34%	1.75%
	FTO-CDA1	0.00%	2.88%	1.10%	0.00%	1.64%	2.79%	3.07%	0.00%	0.00%	2.41%	0.00%	2.40%
	RF-CDA1	0.00%	0.00%	0.00%	2.02%	2.91%	0.00%	0.00%	0.00%	0.00%	0.00%	0.00%	93.58%

Supplemental Table 7. The C-to-T editing efficiency of CDA1, CDA1-DBD, CDA1-HNHN and CDA1-HNHG in potato hairy roots

Target sites	CBE constructs	C-to-T editing efficiency in independent transgenic hairy roots											
		1	2	3	4	5	6	7	8	9	10	11	12
StALS1-gRNA1	CDA1	21.25%	0.00%	34.85%	9.47%	0.00%	1.36%	10.19%	44.50%	14.98%	2.87%	75.03%	50.25%
	CDA1-DBD	19.37%	20.13%	22.21%	22.92%	23.61%	21.68%	18.41%	17.44%	17.01%	28.07%	15.20%	21.34%
	CDA1-HNHN	98.64%	51.82%	97.44%	82.73%	0.00%	96.25%	89.82%	96.54%	77.46%	74.44%	83.42%	78.81%
	CDA1-HNHG	100.00%	34.85%	82.52%	97.69%	98.43%	97.99%	20.31%	31.57%	97.05%	48.88%	96.42%	97.20%
StALS2-gRNA1	CDA1	6.25%	0.00%	21.95%	10.45%	0.00%	0.00%	0.00%	7.20%	18.31%	49.54%	46.32%	8.59%
	CDA1-DBD	18.58%	21.84%	18.75%	22.80%	25.67%	19.47%	23.92%	14.57%	20.97%	24.62%	16.48%	26.62%
	CDA1-HNHN	98.20%	53.86%	98.24%	98.33%	0.00%	96.66%	87.73%	49.55%	73.99%	76.23%	83.60%	100.00%
	CDA1-HNHG	88.03%	17.46%	82.08%	74.48%	98.33%	82.00%	3.45%	52.29%	97.21%	97.11%	98.88%	98.50%
StEPSPS2-gRNA1	CDA1	72.19%	9.86%	88.91%	8.73%	1.02%	22.58%	97.65%	63.50%	87.67%	31.93%	94.01%	86.30%
	CDA1-DBD	60.74%	48.14%	39.70%	6.77%	44.97%	89.84%	0.00%	5.23%	70.49%	0.00%	3.38%	95.30%
	CDA1-HNHN	0.00%	100.00%	27.32%	100.00%	98.00%	98.40%	88.69%	100.00%	81.00%	60.95%	98.12%	96.72%
	CDA1-HNHG	93.26%	98.96%	1.91%	76.29%	98.24%	80.07%	98.95%	91.14%	97.91%	98.94%	94.06%	97.98%
StEPSPS2-gRNA2	CDA1	29.61%	9.98%	11.18%	9.93%	24.91%	46.24%	3.29%	0.00%	3.59%	15.37%	5.75%	49.96%
	CDA1-DBD	27.40%	17.90%	1.14%	28.12%	95.89%	1.44%	65.79%	96.63%	51.44%	90.58%	0.00%	15.64%
	CDA1-HNHN	91.64%	91.59%	74.67%	90.13%	98.77%	96.92%	82.88%	59.24%	97.55%	71.57%	98.46%	67.29%
	CDA1-HNHG	97.67%	97.41%	91.62%	49.92%	18.99%	97.40%	77.55%	91.05%	97.45%	98.61%	96.85%	98.35%
StGBSS-gRNA1	CDA1	41.11%	5.31%	58.64%	0.00%	0.00%	6.24%	51.52%	44.16%	21.04%	3.88%	46.77%	63.94%
	CDA1-DBD	61.21%	16.14%	0.00%	51.07%	7.67%	53.56%	26.41%	0.00%	38.53%	29.33%	29.96%	35.77%
	CDA1-HNHN	1.99%	100.00%	18.77%	100.00%	98.82%	100.00%	90.07%	100.00%	81.01%	30.60%	100.00%	1.88%
	CDA1-HNHG	77.80%	100.00%	1.09%	86.42%	89.04%	1.31%	84.07%	87.29%	96.45%	92.02%	66.40%	98.84%
StPDS-gRNA1	CDA1	71.52%	1.50%	64.73%	31.31%	1.51%	2.65%	40.24%	65.17%	5.54%	7.84%	68.13%	35.63%
	CDA1-DBD	47.23%	29.35%	30.93%	15.32%	34.60%	39.29%	19.29%	9.77%	10.41%	35.67%	8.18%	12.88%
	CDA1-HNHN	98.67%	97.69%	97.59%	98.33%	1.95%	97.94%	87.92%	98.04%	98.10%	31.63%	88.20%	100.00%
	CDA1-HNHG	100.00%	97.33%	100.00%	98.69%	100.00%	92.74%	62.32%	76.79%	98.19%	96.00%	98.92%	98.91%
StPDS-gRNA2	CDA1	23.12%	90.14%	52.17%	14.61%	19.63%	25.92%	7.04%	0.00%	7.94%	14.25%	8.61%	30.45%
	CDA1-DBD	43.38%	15.05%	23.66%	30.08%	55.45%	2.66%	43.02%	73.58%	6.35%	70.00%	16.23%	11.64%
	CDA1-HNHN	93.42%	93.21%	100.00%	100.00%	100.00%	97.90%	82.99%	98.83%	98.62%	85.25%	100.00%	75.04%
	CDA1-HNHG	97.11%	98.04%	80.39%	32.92%	33.57%	98.71%	86.18%	92.15%	96.35%	97.34%	96.72%	74.89%
StPDS-gRNA3	CDA1	33.00%	2.29%	7.10%	0.00%	55.00%	16.67%	7.14%	2.18%	0.00%	11.67%	1.47%	8.94%
	CDA1-DBD	29.34%	18.61%	0.00%	32.58%	46.62%	0.00%	23.85%	28.43%	3.11%	25.08%	0.00%	5.89%
	CDA1-HNHN	91.15%	91.49%	87.35%	93.51%	97.88%	98.64%	81.62%	46.62%	96.82%	72.29%	77.92%	46.69%
	CDA1-HNHG	44.31%	96.54%	59.13%	27.95%	14.31%	100.00%	3.24%	10.72%	94.90%	86.85%	59.15%	43.88%

Supplemental Table 8. The InDel frequency of CDA1, CDA1-DBD, CDA1-HNHN and CDA1-HNHG in potato hairy roots

Target sites	CBE constructs	The InDel frequency of CBEs in independent transgenic hairy roots											
		1	2	3	4	5	6	7	8	9	10	11	12
StALS1-gRNA1	CDA1	10.06%	0.00%	2.52%	0.00%	0.00%	0.00%	10.19%	0.00%	0.00%	0.00%	0.00%	0.00%
	CDA1-DBD	0.00%	0.00%	0.00%	0.00%	0.00%	0.00%	0.00%	0.00%	0.00%	0.00%	0.00%	0.00%
	CDA1-HNHN	0.00%	1.86%	0.00%	0.00%	0.00%	0.00%	0.00%	0.00%	0.00%	0.00%	0.00%	18.18%
	CDA1-HNHG	0.00%	0.00%	0.00%	0.00%	0.00%	0.00%	0.00%	0.00%	0.00%	0.00%	0.00%	0.00%
StALS2-gRNA1	CDA1	0.00%	0.00%	10.31%	8.16%	0.00%	0.00%	0.00%	0.00%	3.62%	1.07%	2.15%	0.00%
	CDA1-DBD	0.00%	0.00%	0.00%	0.00%	0.00%	0.00%	0.00%	0.00%	0.00%	0.00%	0.00%	0.00%
	CDA1-HNHN	0.00%	0.00%	0.00%	0.00%	0.00%	0.00%	0.00%	0.00%	0.00%	0.00%	0.00%	0.00%
	CDA1-HNHG	0.00%	0.00%	0.00%	0.00%	0.00%	0.00%	0.00%	0.00%	0.00%	0.00%	0.00%	0.00%
StEPSPS2-gRNA1	CDA1	25.01%	0.00%	3.73%	0.00%	0.00%	14.29%	0.00%	5.48%	0.00%	0.00%	0.00%	0.00%
	CDA1-DBD	20.05%	41.04%	0.00%	0.00%	22.36%	2.31%	0.00%	0.00%	7.77%	0.00%	0.00%	0.00%
	CDA1-HNHN	0.00%	0.00%	57.12%	0.00%	0.00%	0.00%	0.00%	0.00%	0.00%	0.00%	0.00%	0.00%
	CDA1-HNHG	1.33%	0.00%	0.00%	0.00%	0.00%	78.38%	0.00%	15.15%	0.00%	0.00%	0.00%	0.00%
StEPSPS2-gRNA2	CDA1	12.00%	8.96%	7.84%	7.02%	11.26%	1.44%	4.24%	1.41%	0.00%	2.64%	1.30%	13.37%
	CDA1-DBD	1.25%	1.79%	4.71%	1.37%	0.00%	0.00%	1.54%	0.00%	0.00%	0.00%	0.00%	3.02%
	CDA1-HNHN	0.00%	0.00%	0.00%	5.93%	0.00%	2.05%	0.00%	40.31%	1.23%	0.00%	0.00%	45.56%
	CDA1-HNHG	0.00%	0.00%	23.24%	12.32%	5.86%	1.27%	0.00%	0.00%	1.08%	0.00%	0.00%	0.00%
StGBSS-gRNA1	CDA1	1.26%	1.23%	2.65%	0.00%	0.00%	0.00%	5.16%	6.77%	3.66%	2.43%	2.22%	0.00%
	CDA1-DBD	0.00%	1.20%	0.00%	0.00%	7.40%	36.79%	0.00%	0.00%	1.86%	0.00%	0.00%	0.00%
	CDA1-HNHN	0.00%	0.00%	4.62%	0.00%	0.00%	0.00%	0.00%	0.00%	0.00%	0.00%	0.00%	0.00%
	CDA1-HNHG	3.65%	0.00%	0.00%	0.00%	3.96%	0.00%	0.00%	28.31%	8.71%	1.44%	37.56%	0.00%
StPDS-gRNA1	CDA1	26.07%	0.00%	14.42%	17.75%	0.00%	0.00%	15.02%	33.53%	45.95%	4.37%	17.27%	11.05%
	CDA1-DBD	3.96%	3.78%	4.79%	5.32%	5.58%	4.27%	0.00%	5.86%	5.04%	5.73%	3.92%	6.26%
	CDA1-HNHN	0.00%	0.00%	0.00%	0.00%	0.00%	0.00%	0.00%	0.00%	0.00%	5.03%	0.00%	0.00%
	CDA1-HNHG	0.00%	0.00%	0.00%	0.00%	0.00%	5.57%	0.00%	10.79%	0.00%	2.08%	0.00%	0.00%
StPDS-gRNA2	CDA1	1.14%	0.00%	0.00%	0.00%	2.62%	0.00%	0.00%	0.00%	0.00%	2.32%	0.00%	0.00%
	CDA1-DBD	18.18%	0.00%	0.00%	2.25%	21.33%	0.00%	0.00%	0.00%	3.37%	0.00%	0.00%	0.00%
	CDA1-HNHN	0.00%	0.00%	0.00%	0.00%	0.00%	0.00%	0.00%	0.00%	0.00%	0.00%	0.00%	2.27%
	CDA1-HNHG	0.00%	0.00%	14.40%	6.65%	24.86%	0.00%	0.00%	0.00%	0.00%	0.00%	0.00%	0.00%
StPDS-gRNA3	CDA1	7.62%	26.42%	7.15%	0.00%	1.24%	1.54%	1.29%	0.00%	0.00%	4.27%	0.00%	2.69%
	CDA1-DBD	1.53%	4.63%	3.61%	17.56%	2.73%	0.00%	4.32%	5.16%	6.29%	5.65%	0.00%	0.00%
	CDA1-HNHN	0.00%	0.00%	0.00%	10.59%	4.74%	0.00%	0.00%	74.67%	0.00%	8.32%	19.19%	51.13%
	CDA1-HNHG	56.22%	4.00%	63.19%	26.15%	7.53%	0.00%	2.66%	0.00%	1.38%	2.75%	49.24%	15.08%

Supplemental Table 9. The C-to-T editing efficiency of Sdd7, Sdd7-HNHN and RF-Sdd7-HNHN in potato hairy roots

Target sites	CBE constructs	C-to-T editing efficiency in independent transgenic hairy roots											
		1	2	3	4	5	6	7	8	9	10	11	12
StALS1-gRNA2	Sdd7	57.82%	0.00%	0.00%	19.73%	17.12%	0.00%	49.54%	54.89%	0.00%	0.00%	61.68%	36.14%
	Sdd7-HNHN	19.27%	66.15%	65.94%	75.30%	23.63%	9.32%	53.48%	82.75%	50.37%	19.11%	20.40%	16.36%
	RF-Sdd7-HNHN	73.76%	61.98%	70.65%	95.52%	73.93%	87.78%	65.72%	69.82%	70.50%	95.43%	78.75%	81.22%
StALS2-gRNA2	Sdd7	40.29%	0.00%	0.00%	19.76%	4.28%	0.00%	39.27%	56.07%	0.00%	0.00%	46.71%	37.41%
	Sdd7-HNHN	67.81%	19.10%	61.70%	67.98%	23.09%	3.96%	83.18%	57.79%	45.55%	11.10%	17.68%	11.98%
	RF-Sdd7-HNHN	67.52%	63.14%	66.30%	98.01%	41.06%	37.14%	64.40%	39.81%	38.58%	96.30%	93.91%	81.49%
StDL1-gRNA1	Sdd7	70.20%	0.00%	0.00%	72.91%	63.36%	2.90%	14.65%	52.98%	0.00%	3.69%	69.25%	72.86%
	Sdd7-HNHN	81.89%	71.88%	98.46%	96.29%	96.37%	63.08%	98.03%	89.06%	93.62%	74.33%	54.72%	29.16%
	RF-Sdd7-HNHN	74.27%	63.46%	79.82%	97.18%	98.28%	93.29%	58.83%	96.28%	97.28%	95.58%	96.71%	98.06%

Supplemental Table 10. The InDel frequency of Sdd7, Sdd7-HNHN and RF-Sdd7-HNHN in potato hairy roots

Target sites	CBE constructs	The InDel frequency of CBEs in independent transgenic hairy roots											
		1	2	3	4	5	6	7	8	9	10	11	12
StALS1-gRNA2	Sdd7	2.45%	0.00%	0.00%	0.00%	0.00%	0.00%	1.83%	9.17%	0.00%	0.00%	0.00%	1.83%
	Sdd7-HNHN	0.00%	0.00%	0.00%	0.00%	0.00%	0.00%	0.00%	0.00%	0.00%	0.00%	0.00%	0.00%
	RF-Sdd7-HNHN	0.00%	0.00%	0.00%	0.00%	0.00%	0.00%	0.00%	0.00%	0.00%	0.00%	0.00%	0.00%
StALS2-gRNA2	Sdd7	4.54%	0.00%	0.00%	0.00%	0.00%	0.00%	0.00%	0.00%	0.00%	0.00%	0.00%	0.00%
	Sdd7-HNHN	0.00%	0.00%	0.00%	0.00%	0.00%	0.00%	0.00%	0.00%	0.00%	0.00%	0.00%	0.00%
	RF-Sdd7-HNHN	0.00%	0.00%	0.00%	0.00%	0.00%	0.00%	0.00%	0.00%	0.00%	0.00%	0.00%	0.00%
StDL1-gRNA1	Sdd7	0.00%	0.00%	1.42%	0.00%	0.00%	0.00%	0.00%	0.00%	0.00%	0.00%	0.00%	0.00%
	Sdd7-HNHN	0.00%	2.89%	0.00%	0.00%	0.00%	1.88%	0.00%	0.00%	0.00%	0.00%	0.00%	5.05%
	RF-Sdd7-HNHN	2.43%	2.76%	0.00%	0.00%	0.00%	2.58%	1.38%	1.98%	1.55%	0.00%	2.15%	1.66%

Supplemental Table 11. The editing efficiency of CDA1 and RF-CDA1 in transgenic potato plants

Base editor	Line	Target gene1	Base editing efficiency	P186 editing	Q184* (stop codon) editing	InDel	Target gene2	Base editing efficiency	P184 editing	Q182* (stop codon) editing	InDel
CDA1	6	*StALS1*	6.76%	6.76%	6.76%	34.54%	*StALS2*	8.60%	8.60%	8.60%	40.06%
CDA1	13	*StALS1*	50.52%	40.91%	46.34%	20.42%	*StALS2*	60.99%	45.39%	55.25%	2.93%
CDA1	14	*StALS1*	58.37%	50.68%	41.63%	0.00%	*StALS2*	53.74%	47.62%	53.74%	0.00%
CDA1	20	*StALS1*	38.79%	35.91%	27.36%	0.00%	*StALS2*	41.19%	41.19%	19.24%	4.94%
CDA1	21	*StALS1*	13.01%	6.60%	6.41%	0.00%	*StALS2*	1.11%	1.11%	0.00%	0.00%
CDA1	24	*StALS1*	69.37%	69.37%	47.43%	0.00%	*StALS2*	57.38%	57.38%	55.33%	39.50%
CDA1	25	*StALS1*	68.58%	43.10%	25.48%	0.00%	*StALS2*	8.87%	6.55%	2.32%	0.00%
CDA1	26	*StALS1*	27.85%	27.85%	0.00%	27.36%	*StALS2*	4.71%	4.71%	0.00%	10.63%
CDA1	34	*StALS1*	46.12%	35.01%	38.00%	15.30%	*StALS2*	66.97%	57.96%	24.61%	12.73%
CDA1	35	*StALS1*	47.12%	39.78%	34.13%	6.74%	*StALS2*	27.18%	24.80%	10.49%	49.60%
CDA1	36	*StALS1*	20.03%	14.16%	17.81%	30.32%	*StALS2*	16.21%	14.47%	10.42%	49.57%
CDA1	37	*StALS1*	0.00%	0.00%	0.00%	0.00%	*StALS2*	0.00%	0.00%	0.00%	0.00%
CDA1	38	*StALS1*	57.39%	52.19%	45.33%	28.66%	*StALS2*	39.90%	34.53%	27.41%	0.00%
CDA1	44	*StALS1*	39.35%	21.76%	33.98%	30.74%	*StALS2*	85.43%	79.96%	83.82%	6.43%
CDA1	45	*StALS1*	0.00%	0.00%	0.00%	0.00%	*StALS2*	0.00%	0.00%	0.00%	0.00%
CDA1	46	*StALS1*	2.09%	0.00%	0.00%	0.00%	*StALS2*	1.75%	0.00%	0.00%	0.00%
CDA1	47	*StALS1*	60.79%	58.55%	35.35%	26.90%	*StALS2*	49.12%	47.81%	46.85%	42.58%
CDA1	51	*StALS1*	30.08%	24.73%	22.52%	0.00%	*StALS2*	29.78%	22.13%	29.78%	0.00%
CDA1	52	*StALS1*	1.60%	0.00%	0.00%	0.00%	*StALS2*	1.65%	0.00%	0.00%	0.00%
CDA1	54	*StALS1*	0.00%	0.00%	0.00%	0.00%	*StALS2*	0.00%	0.00%	0.00%	0.00%
CDA1	55	*StALS1*	0.00%	0.00%	0.00%	0.00%	*StALS2*	0.00%	0.00%	0.00%	0.00%
CDA1	59	*StALS1*	0.00%	0.00%	0.00%	0.00%	*StALS2*	0.00%	0.00%	0.00%	0.00%
CDA1	60	*StALS1*	42.21%	25.71%	23.15%	7.17%	*StALS2*	23.71%	18.78%	11.12%	14.48%
CDA1	62	*StALS1*	0.00%	0.00%	0.00%	0.00%	*StALS2*	2.01%	0.00%	0.00%	0.00%
CDA1	63	*StALS1*	51.33%	30.39%	22.73%	1.71%	*StALS2*	62.96%	37.75%	16.45%	0.00%
CDA1	66	*StALS1*	1.93%	0.00%	0.00%	0.00%	*StALS2*	2.21%	0.00%	0.00%	0.00%
CDA1	70	*StALS1*	0.00%	0.00%	0.00%	0.00%	*StALS2*	0.00%	0.00%	0.00%	0.00%
CDA1	71	*StALS1*	2.09%	0.00%	0.00%	0.00%	*StALS2*	3.38%	0.00%	0.00%	0.00%
CDA1	73	*StALS1*	0.00%	0.00%	0.00%	0.00%	*StALS2*	0.00%	0.00%	0.00%	0.00%
CDA1	74	*StALS1*	2.29%	0.00%	0.00%	0.00%	*StALS2*	0.00%	0.00%	0.00%	0.00%
CDA1	75	*StALS1*	48.02%	15.55%	18.25%	27.45%	*StALS2*	34.08%	20.86%	25.50%	33.69%
CDA1	76	*StALS1*	59.51%	16.29%	12.61%	27.74%	*StALS2*	53.92%	41.62%	39.61%	17.00%
CDA1	77	*StALS1*	63.67%	15.31%	35.84%	26.97%	*StALS2*	71.90%	54.60%	39.93%	8.76%
CDA1	78	*StALS1*	58.27%	18.69%	25.94%	25.99%	*StALS2*	61.58%	40.65%	34.77%	13.36%
CDA1	79	*StALS1*	71.14%	26.88%	16.86%	28.85%	*StALS2*	61.87%	45.34%	44.95%	14.75%
RF-CDA1	1	*StALS1*	56.75%	40.99%	11.47%	4.39%	*StALS2*	70.14%	54.50%	35.68%	0.00%
RF-CDA1	2	*StALS1*	45.74%	32.22%	11.50%	5.50%	*StALS2*	24.24%	15.15%	14.97%	38.31%
RF-CDA1	3	*StALS1*	62.80%	41.03%	20.98%	28.30%	*StALS2*	51.96%	32.82%	2.75%	38.55%
RF-CDA1	4	*StALS1*	18.06%	9.91%	12.28%	14.94%	*StALS2*	23.53%	16.43%	3.25%	55.90%
RF-CDA1	5	*StALS1*	52.64%	7.69%	27.44%	25.08%	*StALS2*	34.45%	31.81%	6.50%	41.51%
RF-CDA1	6	*StALS1*	13.76%	9.21%	12.18%	30.04%	*StALS2*	32.62%	12.35%	17.16%	8.45%
RF-CDA1	7	*StALS1*	58.70%	42.55%	32.08%	1.45%	*StALS2*	60.86%	20.01%	35.62%	7.15%
RF-CDA1	8	*StALS1*	19.16%	11.72%	14.47%	67.53%	*StALS2*	57.87%	16.63%	33.12%	11.47%
RF-CDA1	9	*StALS1*	14.19%	6.10%	11.23%	0.00%	*StALS2*	33.19%	26.17%	2.77%	0.00%
RF-CDA1	10	*StALS1*	39.20%	34.81%	6.47%	30.36%	*StALS2*	39.12%	16.41%	39.12%	56.68%
RF-CDA1	11	*StALS1*	52.10%	26.28%	49.28%	18.78%	*StALS2*	23.56%	7.82%	16.08%	40.49%
RF-CDA1	12	*StALS1*	49.46%	31.97%	33.04%	28.09%	*StALS2*	70.42%	33.81%	30.31%	6.25%
RF-CDA1	13	*StALS1*	58.58%	30.74%	50.27%	24.67%	*StALS2*	81.83%	49.17%	0.00%	15.05%
RF-CDA1	15	*StALS1*	53.82%	33.17%	43.21%	22.72%	*StALS2*	74.52%	40.00%	10.96%	12.61%
RF-CDA1	16	*StALS1*	7.28%	4.58%	4.70%	0.00%	*StALS2*	6.12%	6.12%	0.00%	0.00%
RF-CDA1	17	*StALS1*	39.85%	30.84%	3.80%	11.27%	*StALS2*	27.66%	0.00%	0.00%	69.51%
RF-CDA1	18	*StALS1*	40.32%	10.88%	3.04%	6.56%	*StALS2*	9.68%	0.00%	0.00%	69.37%
RF-CDA1	19	*StALS1*	40.46%	32.34%	35.19%	26.62%	*StALS2*	79.10%	51.17%	0.00%	16.58%
RF-CDA1	20	*StALS1*	39.08%	31.16%	31.69%	32.04%	*StALS2*	79.77%	44.48%	0.00%	17.28%
RF-CDA1	21	*StALS1*	51.29%	25.00%	42.53%	22.41%	*StALS2*	27.91%	0.00%	8.64%	47.51%
RF-CDA1	22	*StALS1*	38.55%	28.87%	30.32%	30.32%	*StALS2*	83.64%	52.02%	0.00%	16.36%
RF-CDA1	23	*StALS1*	46.35%	35.73%	36.20%	27.22%	*StALS2*	88.41%	54.93%	3.26%	11.59%
RF-CDA1	24	*StALS1*	41.86%	31.06%	34.82%	24.78%	*StALS2*	68.38%	45.34%	0.00%	29.48%
RF-CDA1	28	*StALS1*	44.88%	29.81%	33.33%	25.48%	*StALS2*	86.16%	47.23%	0.00%	9.88%
RF-CDA1	29	*StALS1*	55.13%	27.96%	29.80%	32.06%	*StALS2*	83.92%	52.10%	0.00%	16.08%
RF-CDA1	30	*StALS1*	32.74%	22.46%	22.46%	48.61%	*StALS2*	100.00%	61.04%	0.00%	0.00%
RF-CDA1	31	*StALS1*	36.99%	3.52%	29.27%	45.80%	*StALS2*	85.54%	51.12%	0.00%	14.46%
RF-CDA1	32	*StALS1*	42.77%	30.53%	36.73%	29.50%	*StALS2*	85.11%	52.77%	0.00%	14.88%
RF-CDA1	33	*StALS1*	56.95%	30.04%	54.40%	24.60%	*StALS2*	80.96%	50.33%	0.00%	19.04%
RF-CDA1	34	*StALS1*	56.23%	29.09%	47.90%	25.84%	*StALS2*	82.84%	51.34%	3.35%	17.16%
RF-CDA1	35	*StALS1*	50.20%	6.11%	24.97%	41.04%	*StALS2*	20.64%	5.92%	6.72%	43.36%
RF-CDA1	36	*StALS1*	42.47%	28.04%	30.20%	29.12%	*StALS2*	76.40%	50.73%	4.50%	16.55%
RF-CDA1	37	*StALS1*	50.57%	2.47%	0.00%	27.01%	*StALS2*	34.06%	31.03%	0.00%	25.66%
RF-CDA1	38	*StALS1*	20.05%	13.06%	10.94%	33.12%	*StALS2*	27.05%	27.05%	47.18%	64.62%
RF-CDA1	39	*StALS1*	44.32%	31.29%	34.15%	23.99%	*StALS2*	76.10%	45.04%	3.81%	17.67%
RF-CDA1	40	*StALS1*	24.94%	0.00%	24.94%	0.00%	*StALS2*	3.75%	0.00%	3.75%	0.00%
RF-CDA1	41	*StALS1*	45.12%	16.34%	35.69%	11.37%	*StALS2*	18.73%	7.30%	7.93%	41.59%
RF-CDA1	42	*StALS1*	79.58%	76.81%	51.21%	0.00%	*StALS2*	95.81%	23.83%	95.81%	0.00%
RF-CDA1	43	*StALS1*	79.05%	70.19%	49.48%	0.00%	*StALS2*	97.75%	78.29%	42.96%	0.00%
RF-CDA1	44	*StALS1*	55.70%	36.75%	24.86%	25.64%	*StALS2*	78.64%	0.00%	0.00%	17.78%
RF-CDA1	45	*StALS1*	35.00%	0.00%	35.00%	0.00%	*StALS2*	2.59%	0.00%	2.59%	0.00%
RF-CDA1	46	*StALS1*	51.97%	3.73%	28.49%	9.07%	*StALS2*	73.16%	41.82%	47.94%	5.24%

Supplemental Table 12. The editing efficiency of Sdd7, Sdd7-HNHN and RF-Sdd7-HNHN in transgenic potato plants

Base editor	Line	Target gene 1	Base editing efficiency	P186 editing	Q184* (stop codon) editing	InDel	Target gene 2	Base editing efficiency	P184 editing	Q182* (stop codon) editing	InDel
Sdd7	1	*StALS1*	1.32%	1.32%	0.00%	0.00%	*StALS2*	0.00%	0.00%	0.00%	0.00%
Sdd7	2	*StALS1*	0.00%	0.00%	0.00%	0.00%	*StALS2*	0.00%	0.00%	0.00%	0.00%
Sdd7	3	*StALS1*	1.87%	1.87%	0.00%	0.00%	*StALS2*	0.00%	0.00%	0.00%	0.00%
Sdd7	4	*StALS1*	0.00%	0.00%	0.00%	0.00%	*StALS2*	0.00%	0.00%	0.00%	0.00%
Sdd7	5	*StALS1*	1.80%	1.80%	0.00%	0.00%	*StALS2*	0.00%	0.00%	0.00%	0.00%
Sdd7	6	*StALS1*	27.62%	27.62%	0.00%	0.00%	*StALS2*	24.32%	24.32%	14.23%	0.00%
Sdd7	7	*StALS1*	0.00%	0.00%	0.00%	0.00%	*StALS2*	0.00%	0.00%	0.00%	0.00%
Sdd7	8	*StALS1*	0.00%	0.00%	0.00%	0.00%	*StALS2*	0.00%	0.00%	0.00%	0.00%
Sdd7-HNHN	1	*StALS1*	46.28%	46.28%	0.00%	0.00%	*StALS2*	2.28%	2.28%	0.00%	0.00%
Sdd7-HNHN	2	*StALS1*	4.32%	4.32%	0.00%	0.00%	*StALS2*	6.19%	6.19%	0.00%	0.00%
Sdd7-HNHN	3	*StALS1*	54.25%	54.25%	23.38%	0.00%	*StALS2*	45.13%	45.13%	0.00%	0.00%
Sdd7-HNHN	4	*StALS1*	51.18%	51.18%	0.00%	0.00%	*StALS2*	55.87%	55.87%	0.00%	0.00%
Sdd7-HNHN	5	*StALS1*	4.02%	4.02%	0.00%	0.00%	*StALS2*	37.60%	37.60%	0.00%	0.00%
Sdd7-HNHN	6	*StALS1*	49.78%	49.78%	0.00%	0.00%	*StALS2*	49.16%	49.16%	0.00%	0.00%
Sdd7-HNHN	7	*StALS1*	7.34%	7.34%	0.00%	0.00%	*StALS2*	5.68%	5.68%	0.00%	0.00%
Sdd7-HNHN	8	*StALS1*	28.18%	28.18%	0.00%	0.00%	*StALS2*	6.79%	0.00%	6.79%	0.00%
Sdd7-HNHN	9	*StALS1*	7.82%	7.82%	0.00%	0.00%	*StALS2*	2.70%	2.70%	0.00%	0.00%
Sdd7-HNHN	12	*StALS1*	0.00%	0.00%	0.00%	0.00%	*StALS2*	2.98%	0.00%	0.00%	0.00%
Sdd7-HNHN	13	*StALS1*	0.00%	0.00%	0.00%	0.00%	*StALS2*	0.00%	0.00%	0.00%	0.00%
Sdd7-HNHN	14	*StALS1*	0.00%	0.00%	0.00%	0.00%	*StALS2*	0.00%	0.00%	0.00%	0.00%
Sdd7-HNHN	15	*StALS1*	0.00%	0.00%	0.00%	0.00%	*StALS2*	4.03%	4.03%	0.00%	0.00%
Sdd7-HNHN	16	*StALS1*	0.00%	0.00%	0.00%	0.00%	*StALS2*	0.00%	0.00%	0.00%	0.00%
Sdd7-HNHN	17	*StALS1*	6.68%	6.68%	0.00%	0.00%	*StALS2*	6.04%	6.04%	0.00%	0.00%
Sdd7-HNHN	19	*StALS1*	0.00%	0.00%	0.00%	0.00%	*StALS2*	0.00%	0.00%	0.00%	0.00%
Sdd7-HNHN	20	*StALS1*	0.00%	0.00%	0.00%	0.00%	*StALS2*	0.00%	0.00%	0.00%	0.00%
Sdd7-HNHN	21	*StALS1*	12.62%	12.62%	0.00%	0.00%	*StALS2*	5.44%	5.44%	0.00%	0.00%
Sdd7-HNHN	22	*StALS1*	4.28%	4.28%	0.00%	0.00%	*StALS2*	5.76%	0.00%	5.76%	0.00%
Sdd7-HNHN	23	*StALS1*	48.18%	48.18%	0.00%	0.00%	*StALS2*	65.40%	65.40%	0.00%	0.00%
RF-Sdd7-HNHN	1	*StALS1*	12.37%	10.40%	0.00%	0.00%	*StALS2*	82.37%	0.00%	82.37%	0.00%
RF-Sdd7-HNHN	2	*StALS1*	4.74%	4.74%	0.00%	0.00%	*StALS2*	0.00%	0.00%	0.00%	34.13%
RF-Sdd7-HNHN	3	*StALS1*	14.39%	14.39%	0.00%	0.00%	*StALS2*	35.15%	17.01%	3.02%	2.84%
RF-Sdd7-HNHN	4	*StALS1*	24.45%	6.92%	24.45%	22.09%	*StALS2*	52.47%	48.57%	3.90%	0.00%
RF-Sdd7-HNHN	5	*StALS1*	17.10%	17.10%	0.00%	0.00%	*StALS2*	17.08%	17.08%	0.00%	0.00%
RF-Sdd7-HNHN	6	*StALS1*	94.33%	94.33%	0.00%	0.00%	*StALS2*	95.07%	95.07%	0.00%	0.00%
RF-Sdd7-HNHN	7	*StALS1*	94.17%	94.17%	0.00%	0.00%	*StALS2*	100.00%	100.00%	0.00%	0.00%
RF-Sdd7-HNHN	8	*StALS1*	100.00%	100.00%	25.97%	0.00%	*StALS2*	100.00%	100.00%	0.00%	0.00%
RF-Sdd7-HNHN	9	*StALS1*	100.00%	100.00%	0.00%	0.00%	*StALS2*	100.00%	100.00%	0.00%	0.00%
RF-Sdd7-HNHN	10	*StALS1*	91.43%	91.43%	0.00%	0.00%	*StALS2*	83.33%	83.33%	0.00%	0.00%
RF-Sdd7-HNHN	11	*StALS1*	24.58%	24.58%	0.00%	0.00%	*StALS2*	8.31%	8.31%	0.00%	0.00%
RF-Sdd7-HNHN	12	*StALS1*	76.38%	76.38%	25.11%	23.62%	*StALS2*	97.30%	97.30%	0.00%	87.39%
RF-Sdd7-HNHN	13	*StALS1*	11.71%	11.71%	0.00%	0.00%	*StALS2*	27.34%	27.34%	0.00%	0.00%
RF-Sdd7-HNHN	14	*StALS1*	71.64%	71.64%	3.71%	0.00%	*StALS2*	69.79%	69.79%	11.93%	0.00%

Supplemental Table 13. Detection of off-target effects in *Stals* mutants generated by RF-Sdd7-HNHN

Predicted off-target sites	Possible off-target spacer with PAM	Chromosome	Position	Direction	Mismatches	Edited
OFF1	CAAtTGtgGAGGcGGATGATGGG	chr01	82135552	+	4	No
OFF2	aAgGTctCGAGGAGGATGATTGG	chr04	52418613	-	4	No
OFF3	aAgGTcCCGgGGAGGATGATTGG	chr08	47992790	-	4	No
OFF4	gAAGTGagGAGGAGGATGAgGGG	chr09	64080346	+	4	No
OFF5	CAAacGCaGAGGAGGcTGATGGG	chr11	9218154	+	4	No

Supplemental Table 14. Primers used in this study

Supplemental Table 14A. Primers used for target sequencing

Oligo Name	Sequence (5' to 3')	Target	Sequencing
StALS-P186-F	CATCAGGCTTTGACACGTTC	StALS-P186	Sanger
StALS-P186-R	CGCTTCACGAACAACCCTAG		
StALS01-F	ggagtgagtacggtgtgcCCCATAACGACGTTTCTGAAC	StALS-gRNA1	NGS
StALS01-R	gagttggatgctggatggGTCAAAGCCTGATGAATCTCC		
StALS02-F	ggagtgagtacggtgtgcGTCTTGCGGATGCTTTGTTG	StALS-gRNA2	NGS
StALS02-R	gagttggatgctggatggCGCTTCACGAACAACCCTAG		
StDL101-F	gagttggatgctggatggCGCTTCACGAACAACCCTAG	StDL1-gRNA1	NGS
StDL101-R	ggagtgagtacggtgtgcATGGTACTGGTGGAAATTGGA		
StGBSS01-F	ggagtgagtacggtgtgcTGCAGCAAGCTTTTCATTTC	StGBSS-gRNA1	NGS
StGBSS01-R	gagttggatgctggatggGCTCCCATATGGAATCCAGT		
StGBSS02-F	ggagtgagtacggtgtgcCGGACATCGGGTAATGACAA	StGBSS-gRNA2	NGS
StGBSS02-R	gagttggatgctggatggTGCTGTCTCCAACTTTGACC		
StEPSPS201-F	ggagtgagtacggtgtgcTGTCTGGATCCATTAGCAGC	StEPSPS2-gRNA1	NGS
StEPSPS201-R	gagttggatgctggatggCCAAATCGCTCCATCAACTTC		
StEPSPS202-F	ggagtgagtacggtgtgcTGATTCTTATGCTGCCCTTGG	StEPSPS2-gRNA2	NGS
StEPSPS202-R	gagttggatgctggatggCAAGTGGGGTGTATGCAGAC		
StPDS01-F	ggagtgagtacggtgtgcCTTACTTACGCAGTCACCAAG	StPDS-gRNA1	NGS
StPDS01-R	gagttggatgctggatggCCAGTCTCGTACCAATCTCC		
StPDS02-F	ggagtgagtacggtgtgcCTTTTAAGTTGGGGCTTACCC	StPDS-gRNA2	NGS
StPDS02-R	gagttggatgctggatggCAAAGCGGCTAAATTCTCCTG		
StPDS03-F	ggagtgagtacggtgtgcGACCCTCTGACATAACTGGC	StPDS-gRNA3	NGS
StPDS03-R	gagttggatgctggatggGCTAGTTCCTTCATCGTGGC		
OFF1-F	ggagtgagtacggtgtgcTACAAACCTCAATGCAACACG	OFF1	NGS
OFF1-R	gagttggatgctggatggCAACCCTTAGCCCAATGCTTA		
OFF2-F	ggagtgagtacggtgtgcGAACAGAATTCAAACAAGCTG	OFF2	NGS
OFF2-R	gagttggatgctggatggTTGTCGTGCTATCTTTGACTC		
OFF3-F	ggagtgagtacggtgtgcAAAGACCTTCAGACTCTCTAG	OFF3	NGS
OFF3-R	gagttggatgctggatggGGGACAGTCAAACTAAACTC		
OFF4-F	ggagtgagtacggtgtgcCCTCCTCTTTTCTCTCTTGT	OFF4	NGS
OFF4-R	gagttggatgctggatggGACATCTACTCTCTACACTCC		
OFF5-F	ggagtgagtacggtgtgcGAGGTGCCTTGATGATACTTC	OFF5	NGS
OFF5-R	gagttggatgctggatggATGCATTTCACTTGACCACA		

Supplemental Table 14B. Primers for vector construction and RT-qPCR

Oligo Name	Sequence (5' to 3')	Purpose
Anc689-F	CGAAGAAGAAGCGCAAGGTCAGCAGTGAAACCGGACCAGT	Amplify Anc689 APOBEC1
Anc689-R	CCGCCGCTAGAGCCACCAGAGAGCTCTTTAAGGCCTGTAGCCCACA	
evoFERNY-F	CGAAGAAGAAGCGCAAGGTCTTCGAGCGCAACTACGACCC	Amplify evoFERNY
evoFERNY-R	CCGCCGCTAGAGCCACCAGAGAGCTCAAGTTTGAGGGAGTACTGCT	
Sdd7-F	CGAAGAAGAAGCGCAAGGTCGAAGGTGGAGGACCAGGTGC	Amplify Sdd7
Sdd7-R	CCGCCGCTAGAGCCACCAGAGAGCTCTGACCACCAAGGCCTAGCTC	
CDA1-F	CGAAGAAGAAGCGCAAGGTCACAGACGCTGAATATGTTAG	Amplify CDA1
CDA1-R	CCGCCGCTAGAGCCACCAGAGAGCTCTACAGCCGGGCTTTTGGTTG	
CDA1-m1-F	TTAAGAAGCAGTTCTCTAATAACAAAAAGTC	Amplify evoCDA1
CDA1-m1-R	GACTTTTTGTTATTAGAGAACTGCTTCTTAA	
CDA1-m2-F	CAAGATTTGGGTTTGCAAACTTT	
CDA1-m2-R	AAAGTTTGCAAACCCAAATCTTG	
CDA1-m3-F	GAGTATCATGTTCCAGGTCAAAAT	
CDA1-M3-R	ATTTTGACCTGGAACATGATACTC	
DBD-BF1	AACCAAAAGCCCGGCTGTAGAGCTCTCTGGAGGATCTTCTGGTGG	Amplify DBD
DBD-R	CCGCCGCTAGAGCCACCAGATTGAAGGAGCTTGTCCAGCT	
HN1-R	CCGCCGCTAGAGCCACCAGAATCAGATTTAGCTTCCTTTT	Amplify HN1
HN1-tF	GGGCGACTCCGGCGGCAGCGGTACCATGCCTAAGAGAAAAGTTTC	
TMV-F1	CTAGAGTATTTTTACAACAATTACCAACAACAACAAACAACAAACAACATTACAATTAC	Synthesize TMVΩ
TMV-R1	TGTAATTGTAAATAGTAATTGTAATGTTGTTTGTTGTTTGTTGTTGTTGGTAATTGTTG	
TMV-F2	TGTTTCTCTCACCACAGCCTCTAGAGTATTTTTACAACAA	
TMV-R2	CAGCTTTTTTGTACAAACTTGTTGTAATTGTAAATACTAATT	
RPS-F	AAACCAATAGCTTGCATGCCTGCAGGCTCAACTTTTGATTCGCTATTTG	Amplify AtRPS5Ap
RPS-R	TAATTGTTGTAAAAATACTCTAGAGGCTGTGGTGACAGAAACAG	
FTO-35Sp-F1	CGGTATCGGATCCCCGGCTGCAGGTCAACATGGTG	Amplify hFTO
FTO-35Sp-R	GCTGTAGGAGTTCTTTTCATCCTAGATTACTCGAGTATCG	
FTO-F1	ATGAAAAGAACTCCTACAGC	
FTO-R1	TTATGGTTTAGCCTCCAAAAG	
FTO-NOS-F	CTTTTGGAGGCTAAACCATAAGATCGTTCAAACATTTGGC	
FTO-NOS-R	ACCATGTTGGTGAGACTTTGGATCTAGTAACATAGATGAC	
Alsg1-F	CGCCCCTGACGAACCCAGAAGTTTAAGAGCTATGCTGG	Primers for T1 construct
Epsg1-GF	gcGGTCTCcTCCTAAAGCCAGGTTTAAGAGCTATGCTGG	
PDSg1-GF	gcGGTCTCcTAGGTAGCTGCAGTTTAAGAGCTATGCTGG	
L2Alsg1-F	gctagagtcgaagtagtgattgCGCCCCTGACGAACCCAGAA	
Epsg1-GR	taGGTCTCaaggagatgtggacaatctcttagtcgactctacc	
PDSg1-GR	taGGTCTCacctaaagggagacaatcactacttcgactctag	
Epsg2-F	TCTCTACCCCACAAAAGCAGGTTTAAGAGCTATGCTGG	Primers for T2 construct
GBSg1-GF	gcGGTCTCcCGATGCACAGATGTTTAAGAGCTATGCTGG	
PDSg2-GF	gcGGTCTCcAATTCTCCAAACGTTTAAGAGCTATGCTGG	
PDSg3-GF	gcGGTCTCcATCAGTCTATGTGTTTAAGAGCTATGCTGG	
L2Epsg2-F	gctagagtcgaagtagtgattgTCTCTACCCCACAAAAGCAG	
GBSg1-GR	taGGTCTCaatcgactggtggcaatctcttagtcgactctacc	
PDSg2-GR	taGGTCTCaaattagggattacaatcactacttcgactctag	
PDSg3-GR	taGGTCTCatgattggggttgcaatctcttagtcgactctacc	
Alsg1-F	CGCCCCTGACGAACCCAGAAGTTTAAGAGCTATGCTGG	Primers for T3 construct
Alsg2-GF	gcGGTCTCcGAGGAGGATGATGTTTAAGAGCTATGCTGG	
GBSg2-GF	gcGGTCTCcACAAAGATGCTTGTTTAAGAGCTATGCTGG	
DLg1-GF	gcGGTCTCcAGCTGGTAAGTTGTTTAAGAGCTATGCTGG	
L2Alsg1-F	gctagagtcgaagtagtgattgCGCCCCTGACGAACCCAGAA	
Alsg2-GR	taGGTCTCacctcggcacttgcaatctcttagtcgactctacc	
GBSg2-GR	taGGTCTCattgtattggtcacaatcactacttcgactctag	
DLg1-GR	taGGTCTCaagctttttgaggcaatctcttagtcgactctacc	
L1tRNA-F	gctagagtcgaagtagtgattgAACAAAGCACCAGTGGTCTA	Universal primers for Golden Gate Assembly
L1Ut-R	gatctggaaaattttgcaaaaaaaaGCACCGACTCGGTGCCAC	
L2tRNA-F	gctagagtcgaagtagtgattg	
L2Ut-R	gatctggaaaattttgcaaaaaaaag	
CDA1-qF	acaaccaggagttgagaggc	qPCR primers for CDA1
CDA1-qR	tgtccctgaggttccacaag	
EIF3e-qF	GAGCACAGGAGAAGATGAAGGAG	qPCR primers for EIF3e
EIF3e-qR	CGTTGGTGAATGCGGCAGTAGG	
